# Peptide-Functionalized Electrospun Meshes for the Physiological Cultivation of Pulmonary Alveolar Capillary Barrier Models in a 3D-Printed Micro-Bioreactor

**DOI:** 10.1021/acsbiomaterials.3c00047

**Published:** 2023-07-04

**Authors:** Puja Jain, Sebastian B. Rauer, Daniel Felder, John Linkhorst, Martin Möller, Matthias Wessling, Smriti Singh

**Affiliations:** † DWI—Leibniz Institute for Interactive Materials, RWTH Aachen University, 52074 Aachen, Germany; ‡ Institute for Chemical Process Engineering, RWTH Aachen University, 52074 Aachen, Germany; § Max Planck Institute for Medical Research, Jahnstraße 29, 69120 Heidelberg, Germany

**Keywords:** co-cultivation, lung model, basement membrane mimic, biofunctionalization, mechanical stimulation

## Abstract

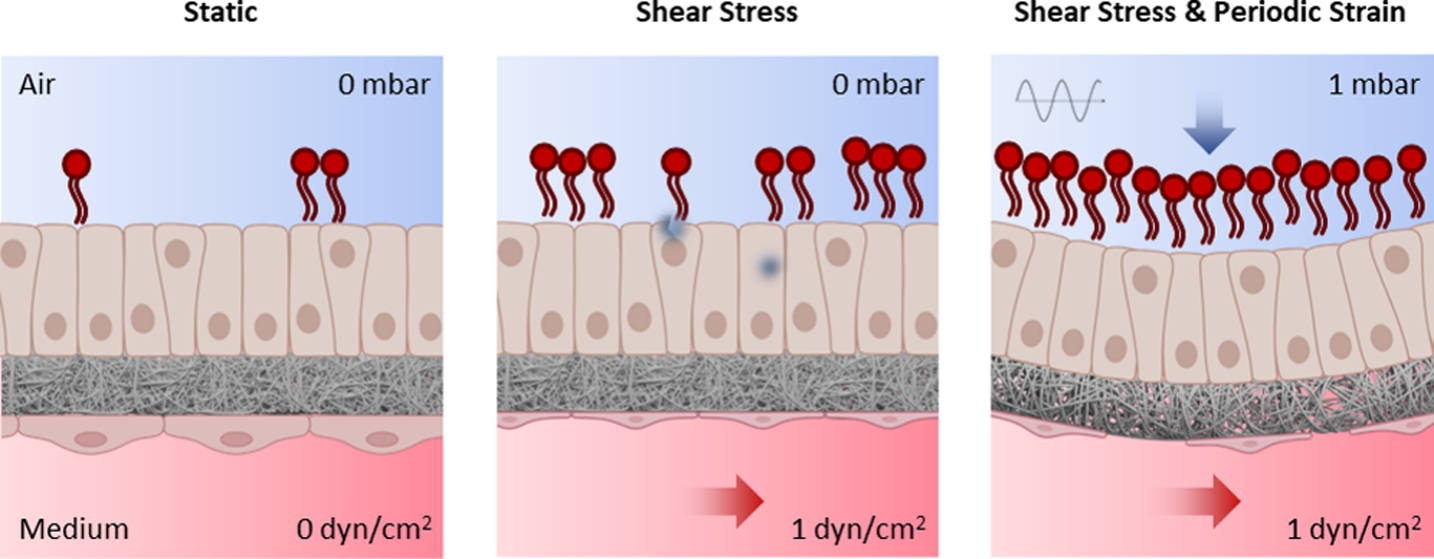

*In vitro* environments that realize biomimetic scaffolds, cellular composition, physiological shear, and strain are integral to developing tissue models of organ-specific functions. In this study, an *in vitro* pulmonary alveolar capillary barrier model is developed that closely mimics physiological functions by combining a synthetic biofunctionalized nanofibrous membrane system with a novel three-dimensional (3D)-printed bioreactor. The fiber meshes are fabricated from a mixture of polycaprolactone (PCL), 6-armed star-shaped isocyanate-terminated poly(ethylene glycol) (sPEG-NCO), and Arg-Gly-Asp (RGD) peptides by a one-step electrospinning process that offers full control over the fiber surface chemistry. The tunable meshes are mounted within the bioreactor where they support the co-cultivation of pulmonary epithelial (NCI-H441) and endothelial (HPMEC) cell monolayers at air–liquid interface under controlled stimulation by fluid shear stress and cyclic distention. This stimulation, which closely mimics blood circulation and breathing motion, is observed to impact alveolar endothelial cytoskeleton arrangement and improve epithelial tight junction formation as well as surfactant protein B production compared to static models. The results highlight the potential of PCL-sPEG-NCO:RGD nanofibrous scaffolds in combination with a 3D-printed bioreactor system as a platform to reconstruct and enhance *in vitro* models to bear a close resemblance to *in vivo* tissues.

## Introduction

*In vitro* models are valuable tools for gaining insights into the physiology and pathophysiology of an organ system at a reproducible scale. These models are engineered to incorporate important features, including cells and their microenvironment, in a simplified manner, such as the popular microfluidic organ-on-chips,^1^ which can be applied to study the influence of environmental factors on cellular behavior. Such platforms open doorways to replace animal models, increase the efficacy and speed of drug development, as well as reduce costs in the study of the respective tissue function.

One such integral organ system to mimic are lung alveoli—the functional unit of the lung. *In vivo*, the lung alveoli are composed of alveolar type I and II cells, a fibrous thin specialized form of basement membrane (BM), microvascular endothelial cells, as well as an interstitial tissue.^2^ The lung alveoli constantly experience physical forces in terms of mechanical stress, which is a force acting per unit area, and strain, which is a measure of local deformation.^3^ On average, the human lung alveoli are subjected to cyclic mechanical strain during inhalation followed by elastic recoil during deflation or expiration.^4^ During normal and deep breathing, the lung alveoli experience a linear strain between 4 and 12%, respectively,^5^ which normally occurs at a rate of 10–20 breaths per minute (0.1–0.3 Hz).^6^ In addition to the periodic distention, the endothelial side of the lung alveoli is further subjected to a constant blood flow, which exerts a fluid shear stress between 0.1 and 80 dyn/cm^2^^7,8^ on the vessel wall, depending on the vessel’s diameter. This dynamic and complex structure of the pulmonary alveolar microenvironment is challenging to mimic in terms of structural and mechanical aspects despite significant advances in the field of tissue engineering and microfluidics. However, since the cellular microenvironment and its multitude of stimuli substantially influence cell behavior and tissue function, its incorporation into *in vitro* models is crucial for creating representative tissues that can be applied for fundamental physiological and pathophysiological research or the development and testing of drugs.

In the context of lung alveoli, numerous studies have already been reported that achieved the incorporation of central pulmonary alveolar features of different complexity into corresponding *in vitro* models. Roshanzadeh et al. developed a bioreactor system for the culture of human epithelial cell monolayers on PDMS membranes to study the uptake of plastic nanoparticles and the alignment of stress fibers under the influence of uniaxial cyclic stretching mimicking human respiration.^9,10^ Cavanaugh et al. fabricated mono-culture *in vitro* models from primary rat lung alveolar type II epithelial cells seeded on silicon-based membranes that were exposed to biaxial cyclic stretch in microtiter plates to investigate the cause of mechanical ventilation-induced lung injury (VILI).^11^ Their results could successfully demonstrate that cyclic stretching of epithelial monolayers for only 2 h already causes increased cell layer permeability due to elevated levels of reactive oxygen species (ROS). Similar studies using a combination of biaxial strain and PDMS-based membrane systems focused on the effects of distention frequency, duration, and amplitude on epithelial cell layer permeability and viability or investigated the influence of sedatives on the epithelium during mechanical ventilation.^12−14^ While all of these studies resulted in valuable scientific contributions regarding central biomedical challenges, the corresponding *in vitro* models are based on membrane systems that do not resemble the fibrous architecture of the natural BM and simplify cyclic stretching by applying either uni- or biaxial instead of physiological triaxial strain.

Since these structural and mechanical cues are fundamental for the development and function of the lung alveoli including proliferation, differentiation, protein production, and activation of integral metabolic pathways, Stucki et al. improved upon the simplified stretching by inventing a microfluidic platform utilizing a micro-diaphragm to indirectly actuate an epithelial-endothelial co-culture model in a triaxial manner.^15,16^ Further advances were made by Zamprogno et al. who employed fibrous collagen-elastin sheets supported on hexagonal gold meshes as BMs for epithelial-endothelial co-cultures to study microvilli formation and model permeability under triaxial strain.^17^ A similar system was also applied by Radiom et al. who immobilized fibrous electrospun gelatin meshes on hexagonal arrays to investigate cell viability and morphology with respect to triaxial cyclic distention.^18^

However, *in vivo* lung alveoli additionally feature a three-dimensional (3D) architecture of complex cellular composition and are exposed to blood flow at the basolateral side of the alveoli. Since cell-to-cell communication and fluid-induced shear stress are both central signals regulating pulmonary alveolar function, *in vitro* models may be further improved by their incorporation. In terms of cellular architecture, bio-printing systems are an upcoming technology that enable the fabrication of more complex layer-by-layer-based *in vitro* models. In this context, Kang et al. developed an all-inkjet drop-on-demand (DOD)-based bio-printing system which they used to fabricate intricate three-layered pulmonary barrier models.^19^ The models can be printed into a commercially available transwell insert and offer a more complex structure featuring an endothelium, a BM, a fibroblast-containing interstitial tissue, and an epithelium. A similar approach using DOD-based bio-printing was applied by Ng et al. who utilized poly(vinylpyrrolidone) (PVP)-based bio-inks for the fabrication of multilayer pulmonary alveolar models.^20^ Additional structural complexity was achieved by Baptista et al. who used a combination of thermoforming and track-etching to produce porous hemispherical polycarbonate (PC) membranes for epithelial cell cultivation.^21^ This principle was further extended by Huang et al. who fabricated GelMA-based inverse 3D opal structures by alginate bead templating which are supplied by continuous medium perfusion and can be exposed to cyclic biaxial strain to study the impact of smoking or pseudo-viral infection.^22^ Although probably one of the most advanced pulmonary alveoli systems, the model does not mimic the sheet-like nature of the BM and therefore cannot provide physiological shear stress to the endothelial side of the *in vitro* model. The lack of relevant fluid shear stress at the endothelium is a general theme in bioreactor systems capable of cyclic pulmonary alveolar model distention and was until now only sparsely addressed, for example, by the famous study of Huh et al., who developed a bioreactor system capable of uniaxial stretch at a maximum shear stress level of 0.2 dyn/cm^2^.^23,24^ However, besides the uniaxial stretch, the system also falls back upon porous PDMS membranes, which again lack the important fibrous architecture of the natural BM.

The present study addresses these limitations by incorporating a functionalized nonwoven mesh supporting the co-cultivation of pulmonary epithelial (NCI-H441) and endothelial (HPMEC) cells in a specialized bioreactor system that enables the exposure of *in vitro* models to independently adjustable blood flow mimicking shear stress and respiration-like triaxial cyclic strain. The fiber meshes are fabricated by electrospinning a polymer solution comprising star-shaped isocyanate-terminated poly(ethylene glycol) (sPEG-NCO), polycaprolactone (PCL), and Arg-Gly-Asp (RGD)-containing peptides resulting in a hydrophilic biofunctionalized PCL-sPEG-NCO:RGD surface closely resembling the topology of natural BM. Covalent bonding of the adhesive peptides to the surface of the fiber mesh is achieved by exploiting the reaction between isocyanate groups and either amine or hydroxyl groups readily available in RGD sequences or other adhesive proteins, opening the possibility to tailor the type, concentration, and composition of surface functionalization. The flexibility and mechanical strength induced by the addition of sPEG-NCO to the fiber mesh allows for a fully elastic response to 15% linear cyclic strain and can be utilized to induce cyclic distention mimicking the physiological breathing motion. The inoculated membranes are mounted in a customized 3D-printed bioreactor, where the epithelial-endothelial co-culture is exposed to an air–liquid interface, continuous fluid shear stress generated by medium perfusion, as well as periodic strain induced by air pressure oscillation. The bioreactor system was applied to investigate the behavior and integrity of both the epithelial and endothelial cells in terms of cell morphology, actin cytoskeleton, tight junction formation, as well as production of surfactant protein B and was compared to the static control specimen. This publication shows the unique combination of fully synthetic tunable nanofibrous meshes closely mimicking the BM topology with a multiuse bioreactor system that enables free-standing cultivation of *in vitro* co-culture models under controllable mechanical stimulation.

## Results and Discussion

### Bioreactor and Process Design

**Figure 1:**
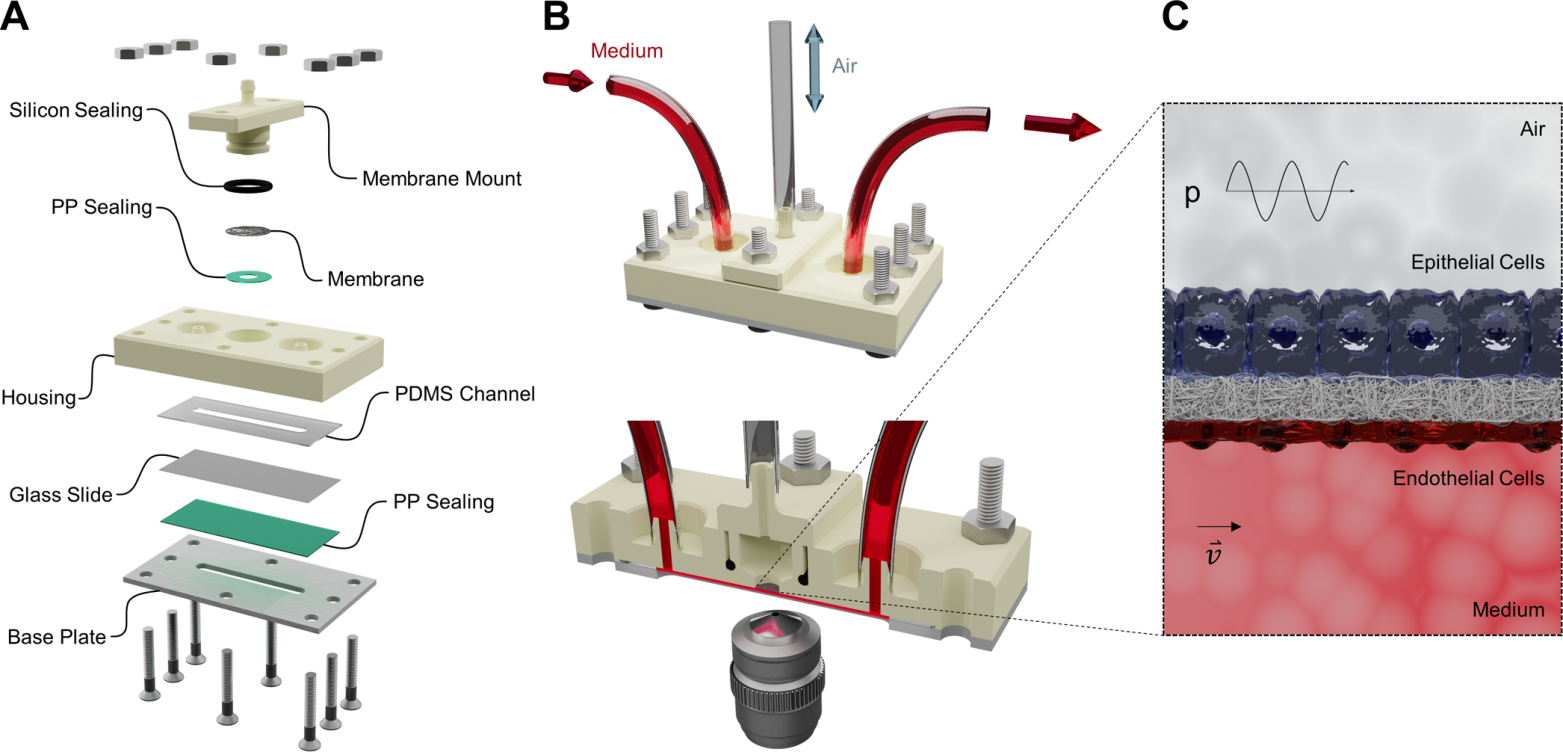
Rendered depiction of the 2-phase micro-physiological 3D-printed bioreactor. (A) Exploded view of the cultivation system, (B) fully assembled bioreactor and cross section, and (C) cell arrangement on a flexible electrospun mesh and an indication of exerted forces.

For the cultivation of pulmonary alveolar capillary barrier models, a 3D-printed micro-physiological 2-phase bioreactor was designed and additively manufactured (Figure 1). The cultivation system was developed to continuously supply cell layers located on a flexible membrane support with aerated medium while also exposing them to physiological conditions such as air–liquid interface, shear stress, and oscillatory strain. Additional goals were bioreactor reusability and the support of live-cell imaging.

Figure 1A presents an exploded view of the bioreactor system, which is divided into an air and a liquid compartment. The air compartment of the bioreactor is composed of a flexible membrane loosely placed onto a hollow membrane mount. The membrane mount, in turn is inserted into a housing component in which the membrane is fixated via press fit. A polypropylene (PP) sealing located in between the membrane and the housing component ensures a tight fit during cultivation, while the silicon sealing ring prevents medium leakage and contamination. Considering that the membrane mount exerts force onto the housing component in the assembled state, the bottom of the membrane mount displays a curved profile to maximize the material thickness between the mount and the flow channel to prevent breakage during operation. A corresponding depiction of the chamber cross section is provided in Figure S1, while detailed part drawings of the 3D-printed housing component are presented in Figures S2 and S3.

The liquid compartment of the bioreactor is composed of a 700 μm thick poly(dimethylsiloxane) (PDMS)-based flow channel, which is placed into a 500 μm deep rectangular groove introduced at the bottom of the housing component. The groove is incorporated for user-friendly alignment and ensures tight sealing by increasing the force exerted by the base plate in the fully assembled state. For live-cell imaging during cultivation, the bottom of the PDMS channel is sealed by a commercially available microscopy slide (60 mm × 24 mm × 170 μm). However, taking into account the distance between the base plate and the membrane, microscopy objectives have to offer at least a working distance of 3 mm for live-cell imaging. This distance could be reduced by milling a conical recess into the aluminum base plate, which would also allow the application of immersion objectives. The thin PP sealing located in between the base plate and the microscopy slide is used to protect the microscopy setup from leakage due to potential glass breakage.

A fully assembled bioreactor is depicted in Figure 1B. Silicon tubings connected to the respective in- and outlets allow for continuous medium perfusion, while a third tubing connected to the membrane mount offers the possibility to change the air compartments’ pressure. Figure 1C shows a cross section of a fully assembled module displaying the two distinct phases of medium and air separated by a flexible electrospun mesh. During cultivation, the two opposing mesh surfaces are occupied by epithelial and endothelial cell monolayers mimicking the apical and basal side of a pulmonary alveolar capillary barrier.

**Figure 2:**
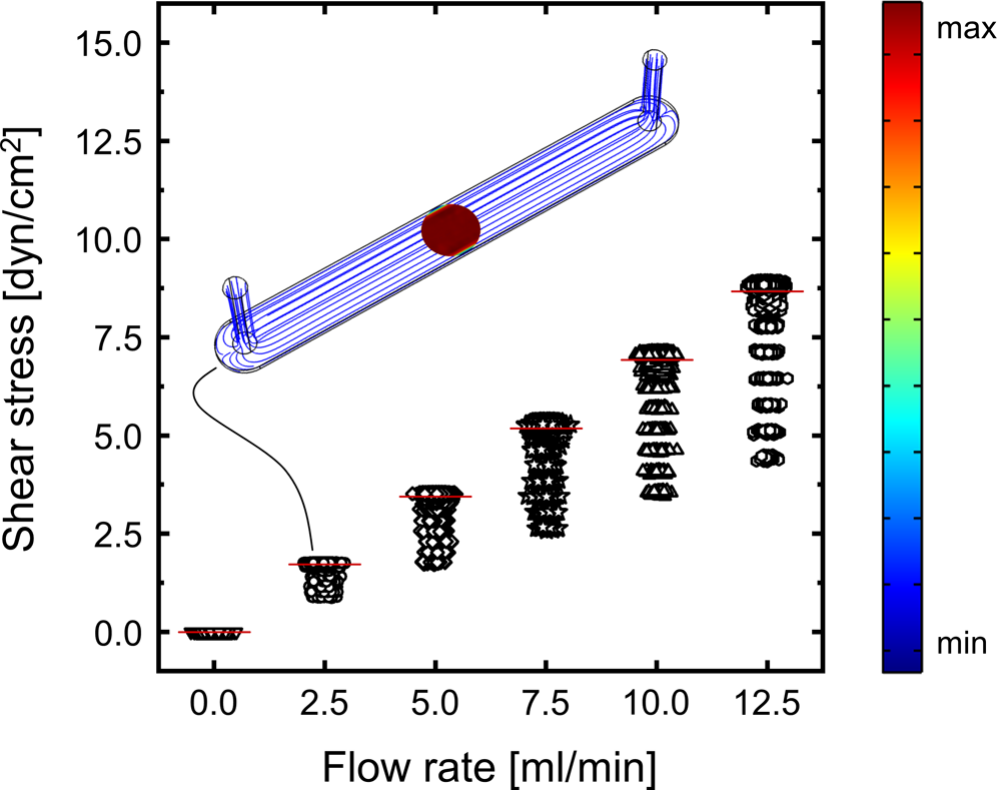
Comsol Multiphysics simulation of the shear stress distribution at different flow rates in a PDMS channel featuring a height of 500 μm and a width of 5 mm. The widespread of data points represents different locations in the chip, where the lower data points depict the wall shear stress, while the upper data points depict the shear stress at the channel center. The red line represents the mean shear stress.

Considering the large body of literature that describes the dependency of cellular behavior on external conditions, capturing key physiological environmental features is crucial for building a representative *in vitro* model.^18,23,25−28^ Concerning the basal side of the pulmonary alveolar capillary barrier, the blood flow-induced shear stress exerted onto the vessel-lining endothelial cells entails a large cascade of cellular responses, such as the reconstitution of the cytoskeleton or the synthesis of growth factors and vasodilative mediators.^29,30^ Investigations regarding the shear stress distribution in the human vascular system resulted in values between 0.1 dyn/cm^2^ up to 80 dyn/cm^2^ depending on the type, location, and size of the vessel.^7,8^ Similar to blood vessels, the shear stress in artificial bioreactor systems is mainly dependent on the correlation between medium flow rate and channel dimensions. For rectangular channel geometries, this correlation can be described by the following equation
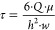
1where τ is the wall shear stress in (N/m^2^), *Q* is the flow rate in (m^3^/s), μ is the dynamic viscosity in (Ns/m^2^), *h* is the channel height in (m), and *w* is the channel width in (m).^31^ This formula can be applied to determine the flow channel dimensions necessary for mimicking physiological shear conditions. For our bioreactor system, PDMS channels were fabricated, displaying an assembled channel height of 500 μm and a channel width of 5 mm, which allows for shear stress levels between 0 and 9 dyn/cm^2^ within a technically feasible flow rate window of 0 to 12.5 mL/min. Figure 2 presents a simulation of the shear stress distribution in dependency of the fluid flow rate within a PDMS channel with Comsol Multiphysics. The area of interest was examined using a uniform 1000 × 1000 point square grid masked by a circular area displaying the size of the membrane. The average shear stress for the respective flow rates is illustrated as red horizontal lines, while the shear stress distribution reaching from the channel center to the wall can be observed as the data point spread. The simulation demonstrates an increasing data point spread with increasing flow rates, which translates into a rising shear stress difference between the wall and the channel center at elevated flow rates. The minimum values of the data point spread present the wall shear stress levels, while the maximum values indicate the center shear stress levels. An exemplary depiction of the shear stress distribution at 2.5 mL/min is observable in the 3D channel depiction, which shows fluid streamlines in blue and the shear stress levels at the cultivation area in color code. A more detailed overview presenting the shear stress and velocity distributions within the channel at different fluid velocities is provided in Figure S4.

**Figure 3:**
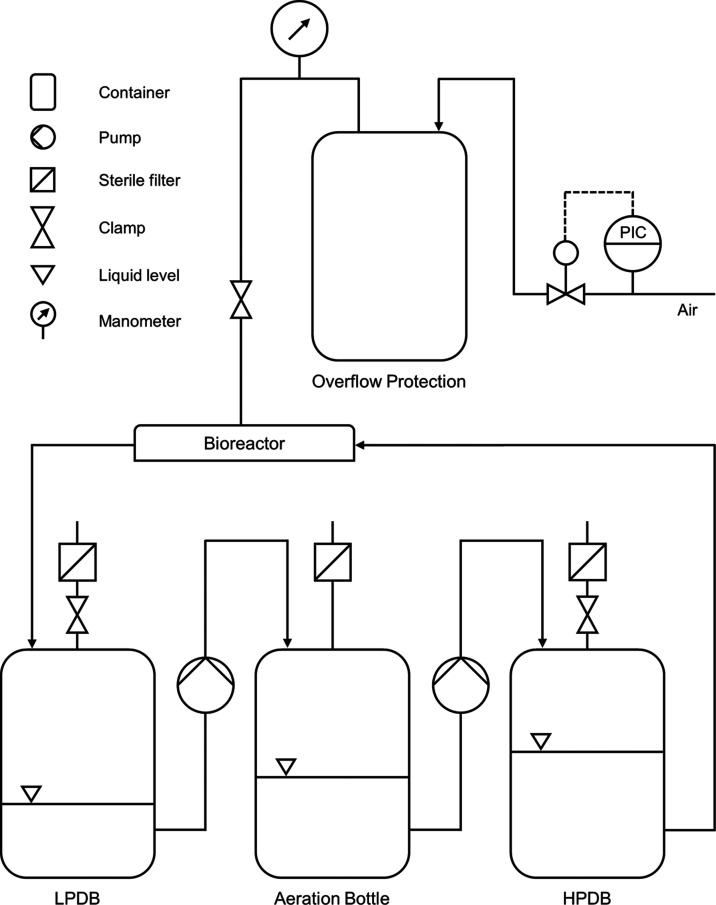
Piping and instrumentation diagram of the circulation setup for co-cultivation of two cell monolayers exposed to fluid shear stress and oscillatory strain. The circulation system comprises two pressure-dampening bottles, one on the low-pressure side (LPDB) and one on the high-pressure side of the pump (HPDB), as well as the aeration bottle for oxygenation.

For continuous bioreactor operation, the system is connected to a medium circulation setup (Figure 3). For cell culture applications, peristaltic pumps are frequently used for medium circulation since all moving pump parts are isolated from the process fluid, thereby preventing cross-contamination during operation.^32−34^ However, peristaltic pumps deliver a pulsatile flow that is dependent on the number of rollers, tube diameter, as well as speed of the pump.^35^ Although this pulsation can, in theory, be used to incorporate oscillatory *in vivo* conditions such as the pulsatile blood flow or the distention of the lung alveoli^34^ into *in vitro* systems, the direct correlation between flow rate and pulsation renders it impossible to run a selective flow rate or shear stress level at a desired pulsing frequency.

To decouple fluid pulsation and flow rate, we incorporated a dampening system into our circulation setup that converts the pulsatile flow of the peristaltic pump into a nonpulsatile one. The effectiveness of our setup is showcased in Videos S5, S6, S7 and S8, which display the response of mounted fiber meshes to a flow rate of 12.5 and 20 mL/min with and without the dampening system, respectively. The dampening system is based on two airtight bottles connected to an aeration bottle via a peristaltic pump. The pump generates a pulsatile medium flow from the aeration bottle into the high-pressure-dampening bottle (HPDB), where the entrapped air pocket absorbs the fluid oscillation. As a result, the medium level within the HPDB rises over time thereby increasing the bottle pressure. This process continues until the over-pressure-induced medium flow rate leaving the HPDB equals the input flow rate. At that point, an equilibrium state is achieved, and both pressure, as well as medium level, remain constant. The medium exiting the HPDB subsequently enters the bioreactor system, where it delivers nutrients as well as oxygen to the cells and exerts shear stress. Considering that both the out- and input of the peristaltic pump have to be decoupled from the bioreactor to prevent uncontrolled pulsation, a second low-pressure-dampening bottle (LPDB) is connected to the circulation setup in between the bioreactor and the peristaltic pump. The peristaltic pump drains medium from the LPDB, thereby decreasing both the medium level as well as the bottle pressure. Like the HPDB, this process continues until the negative pressure-driven medium flow entering the LPDB equals the output flow rate generated by the pump. The medium finally enters the aeration bottle again, where it drips down through an air gap for oxygen uptake. A sterile air filter connects the aeration bottle directly to ambient air replenishing the oxygen continuously.

Hydrostatic pressure constitutes another parameter that significantly influences cellular behavior^36,37^ requiring the regulation to physiological values similar to the previously discussed parameters of pulsation frequency and flow rate. In our circulatory system, pressure is generated within the dampening bottles via the continuous supply and drainage of medium by a peristaltic pump. While the LPDB exhibits a negative pressure in the equilibrium state, the pressure within the HPDB displays a positive value exceeding the atmospheric pressure. In both cases, the absolute pressure value decreases along the tubing system toward the bioreactor due to frictional losses within the tubing system. The Darcy–Weisbach equation describes this correlation for pressure losses in circular pipes
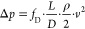
2where *f*_D_ is the Darcy friction (−), *L* is the pipe length in (m), *D* is the pipe diameter in (m), ρ is the fluid density (kg/m^3^), and *v* is the flow velocity (m/s).^38^ Considering a physiological pulmonary alveolar pressure of 0 mbar in the absence of respiratory activity, according to the Darcy–Weisbach equation, the total tubing length between the bioreactor output and the aeration bottle has to be equal to the total tubing length between the aeration bottle and the bioreactor input. In this case, the pressure loss in the system is symmetrical, providing a hydrostatic pressure of 0 mbar at the cultivation area.

Besides the liquid circulation system, the air phase of the bioreactor is additionally connected to a digital pressure indicator, which is applied to determine the base pressure at the cultivation area, and a pressure controller (PIC), which induces oscillatory strain by periodic air pressure regulation around the determined base pressure. The airway connection includes a tubing clamp and an empty container, which are incorporated to protect the PIC in case of a bioreactor malfunction from direct medium contact. The fully assembled setup is depicted in Figure S9. A more detailed description explaining the system’s pressure distribution and a troubleshooting guide is provided in the Supporting Information.

### Characterization of RGD-Functionalized Fiber Meshes

**Figure 4:**
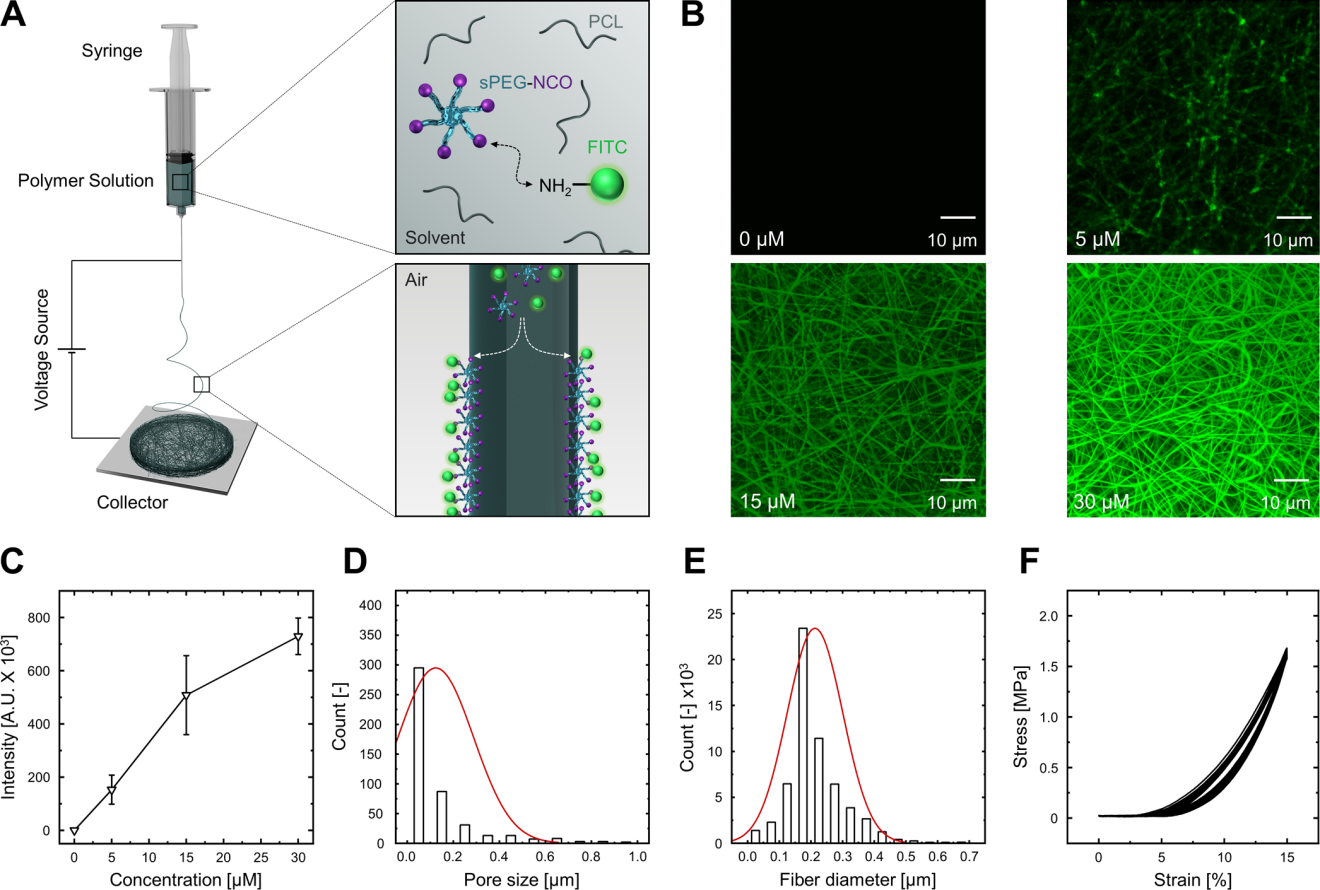
Depiction of the fabrication and characterization of stretchable biofunctionalized nanofibrous electrospun meshes. (A) One-step electrospinning process utilizing a polymer mixture of sPEG-NCO, PCL, and amino-FITC to obtain surface functionalized electrospun meshes. (B) Confocal fluorescence microscopy images of electrospun fiber meshes of different amino-FITC concentrations. (C) Correlation of fluorescence intensity and amino-FITC concentration showing the possibility of tuning the number of adhesive sequences. (D) Pore size distribution observable within fiber meshes. (E) Diameter distribution of individual fibers. (F) Cyclic strain-relaxation at 15% linear strain, a frequency of 0.25 Hz, and 30 cycles. *n* = 3.

The electrospun meshes represent the interwoven and fibrous characteristics of the natural pulmonary alveolar BM and exhibit covalently bound cell-adhesive RGD (H-Arg-Gly-Asp-Cys-OH) peptides to support the long-term adhesion of primary cells. This is achieved in a one-step process by the formation of urethanes or urea bonds between the hydroxyl (−OH) or amine (−NH_2_) groups of the adhesive ligands and the isocyanate end groups (sPEG-NCO) on the electrospun fibers.^39,40^ Surface functionalization occurs during the spinning process when the sPEG-NCO additives surface-segregate on the fibers rendering the meshes hydrophilic (Figure 4A). The surface-segregated additives react with the amine group displaying RGD, resulting in covalently bound surface functionalized fibers. This concept was tested qualitatively by utilizing different concentrations of 6-aminofluorescein (amino-FITC) (0–30 μM) and measuring their corresponding fluorescence intensity. As expected, the fluorescence intensity increased with an increasing amount of amino-FITC and displayed no fluorescence in its absence (Figure 4B,C).

Moreover, the hydrophilic nature of PCL-sPEG-NCO:RGD meshes is supported by the reduced water contact angle measurements of 26 ± 1.2° compared to pure PCL meshes (110 ± 14°) (Figure S10). PCL is a versatile polymer that can be electrospun at various process conditions to fabricate meshes with different fiber characteristics that have an integral influence on cell behavior and function. Hence, the hydrophilic meshes were further assessed in terms of mean pore area and fiber diameter using plugins in ImageJ by analysis of their electron micrograph images. The meshes are characterized by a mean pore area ranging between 0.1 and 0.2 μm^2^ and an equivalent diameter between 200 and 280 nm (Figure 4D) compared to the natural BM (10–130 nm).^41^ Furthermore the fiber diameter of the hydrophilic meshes ranges between 100 and 300 nm, arranged as an interwoven mesh due to layer-by-layer deposition on the collector (Figure 4E). Pulmonary alveoli are functional units of the lung that undergo constant deformation during the breathing process and experience a physiological linear strain between 4 and 12%.^23,42^ This property requires use of polymer blends that can sustain continuous cyclic mechanical strain. Therefore, the mesh was mechanically assessed by applying continuous 30 cyclic strain-relaxations at 15% linear strain and 0.25 Hz using a tensile stretcher. The meshes were tested in the wet state and demonstrated a close-to elastic behavior under cyclic strain exhibiting only a very minor hysteresis or creep as seen in Figure 4F. The mechanical property was further evaluated in terms of tensile stress, resulting in a value of 26.3 ± 2.5 MPa in the wet state. The properties of meshes are thus a close representation of the natural BM in terms of interwoven fibrous structure, presence of ligands for cell adhesion under dynamic stress and strain, as well as mechanical stability under cyclic strains. An overview of all central fiber mesh parameters is presented in Table 1.

**Table 1:** Parameters of PCL-sPEG-NCO:RGD Fiber Meshes

PCL-sPEG-NCO:RGD	properties
mesh type	nonwoven/random mesh
thickness	10 μm
mean pore area	0.1–0.2 μm
equivalent pore diameter	200–280 nm
fiber diameter	100–300 nm
tensile strength	26.3 ± 2.5 MPa
water contact angle	26.0 ± 1.2°

### Distention of Electrospun Fiber Meshes

The lung alveoli constantly experience mechanical strain (expansion/deflation) during breathing or mechanical ventilation, which is described as the change in linear dimension over its initial value (Δ*L*/*L*_0_). This strain is also commonly referred to as stretch or distention.^42^ During each breathing cycle, a negative and positive alveolar pressure of 1 cmH_2_O (∼1 mbar) leads to a triaxial expansion and deflation of the lung alveoli resulting in movement of air inside and out of the alveoli.^43^

**Figure 5:**
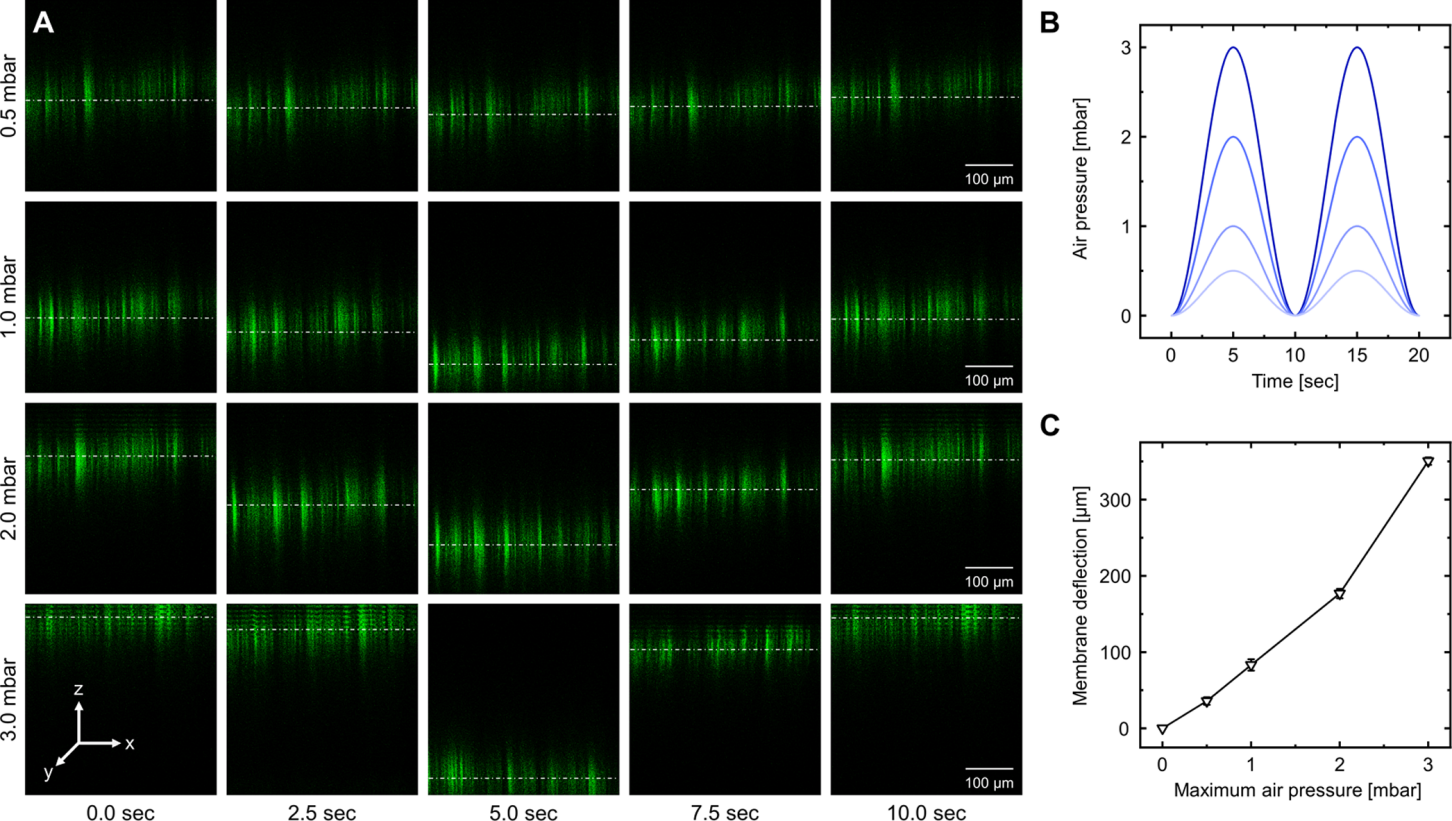
Analysis of membrane distention of electrospun fiber meshes via confocal fluorescence microscopy. (A) Time-lapse series of a membrane section accommodating a fixated layer of Phalloidin-stained epithelial cells at different air pressure amplitudes over a pressure cycle; pressure values present the maximum pressure during oscillation. (B) Idealized pressure progression generated by the pressure controller during the deflection experiment. (C) Distention close to the center of the membrane in dependency of various applied maximum pressures. *n* = 3.

To mimic this cyclic stretch *in vitro* in both amplitude and dimension, a pressure controller was employed that applied a sinusoidal air pressure profile ranging between 0 and 1 mbar. To determine the correlation between applied pressure and mesh deflection, the meshes were seeded with both epithelial and endothelial cells to generate a membrane resistance equal to cultivation conditions. For the investigation of membrane deflection, the cells were stained by applying Phalloidin dye, and xzt confocal microscopy scans were performed close to the membrane center (Figure 5A). The resulting time-lapse images enabled the calculation of mesh displacement over time when exposed to various air pressure amplitudes in sinusoidal cycles, which are depicted in Figure 5B as ideal sinus functions with a frequency of 0.17 Hz. As expected, the mesh displacement increased with increasing magnitude of air pressure from 0 μm up to 350 ± 5 μm, where a physiological pressure of 1 mbar results in a membrane distention of 83 ± 8 μm (Figure 5C). The bioreactor setup, in combination with the mechanical stability of the meshes, enables *in vitro* mimicry of physiological breathing cycles.

### Influence of Mechanical Shear and Oscillatory Strain on the Pulmonary Alveolar Capillary Barrier *In Vitro* Model

**Figure 6:**
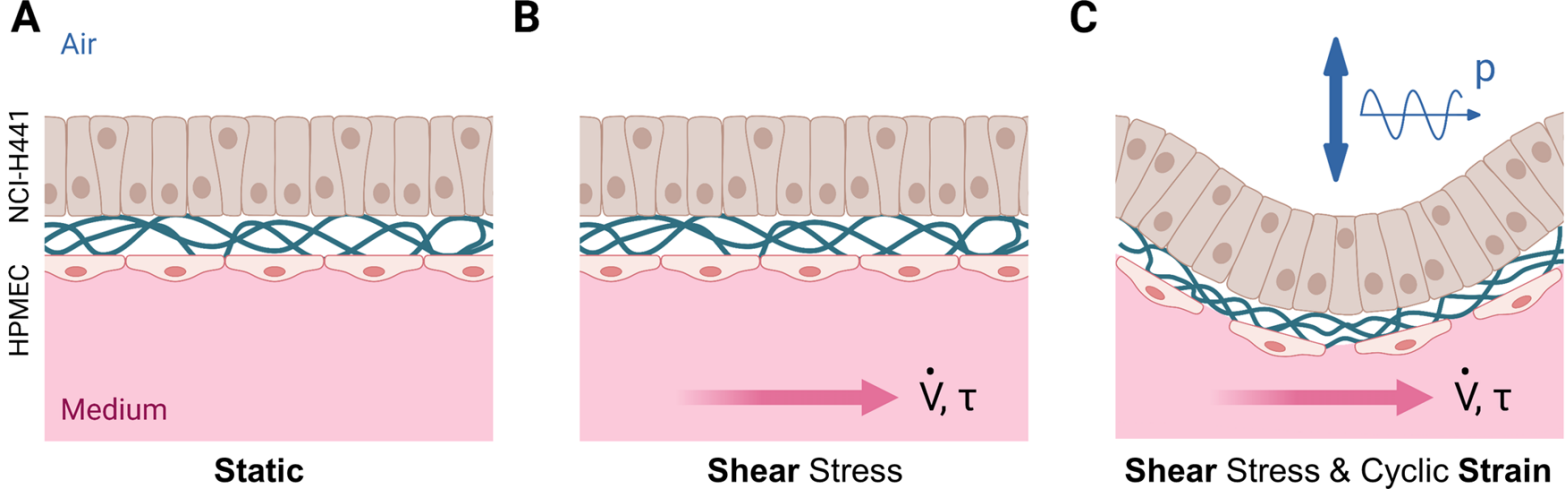
Overview of the experimental conditions studied regarding the co-cultivation of NCI-H441 and HPMEC cells on electrospun PCL-sPEG-NCO:RGD fiber meshes. The experimental conditions include air pressure amplitude p, medium volume flow *V̇*, and shear stress τ. (A) Static cultivation on a transwell insert positioned in a microtiter plate; *p* = 0 mbar, *V̇* = 0.0 mL/min, τ = 0 dyn/cm^2^. (B) Cultivation in the bioreactor system under continuous medium supply exerting shear stress onto the endothelial side of the *in vitro* model; *p* = 0 mbar, *V̇* = 1.1 mL/min, τ = 1 dyn/cm^2^. (C) Cultivation in the bioreactor system under continuous medium supply and air pressure oscillation exerting shear stress and periodic triaxial strain onto the *in vitro* model; *p* = 1 mbar, *V̇* = 1.1 mL/min, τ = 1 dyn/cm^2^. Created with Biorender.com.

The PCL-sPEG-NCO:RGD meshes were used as substrates to co-culture NCI-H441 and HPMEC on opposite sides of the meshes to mimic the functional unit of the lung. NCI-H441 cells were selected as they exhibit similar properties to type II pulmonary alveolar cells in terms of monolayer and barrier properties as well as surfactant protein production.^44^ On attainment of cell confluency on day 4, the meshes were transferred to the bioreactor inserts and introduced to continuous medium flow at the basal side, while the epithelial side was exposed to air. Following 5 days of dynamic cultivation at an air–liquid interface accustoming the cell monolayers to the presence of shear stress, the co-culture models were additionally exposed to triaxial strain by exposing the epithelial side to an oscillatory air pressure of an amplitude of 1 mbar. After 3 h of constant breathing motion at a frequency of 0.17 Hz, air pressure oscillation was terminated and the cultivation was continued under constant medium flow until day 6. Static air–liquid co-culture models without stimulation by shear stress and oscillatory strain were used as control models to study the influence of mechanical forces in terms of cell morphology, actin cytoskeleton, surfactant protein B production, and tight junction formation. Additionally, the cultivation of models under constant fluid shear stress but in the absence of breathing motion was conducted to decouple the two types of mechanical stimuli and evaluate the resulting cell responses independently. A schematic of the experimental conditions investigated and compared in this study is provided in Figure 6.

#### Influence on Epithelial Morphology and Tight Junctions

**Figure 7:**
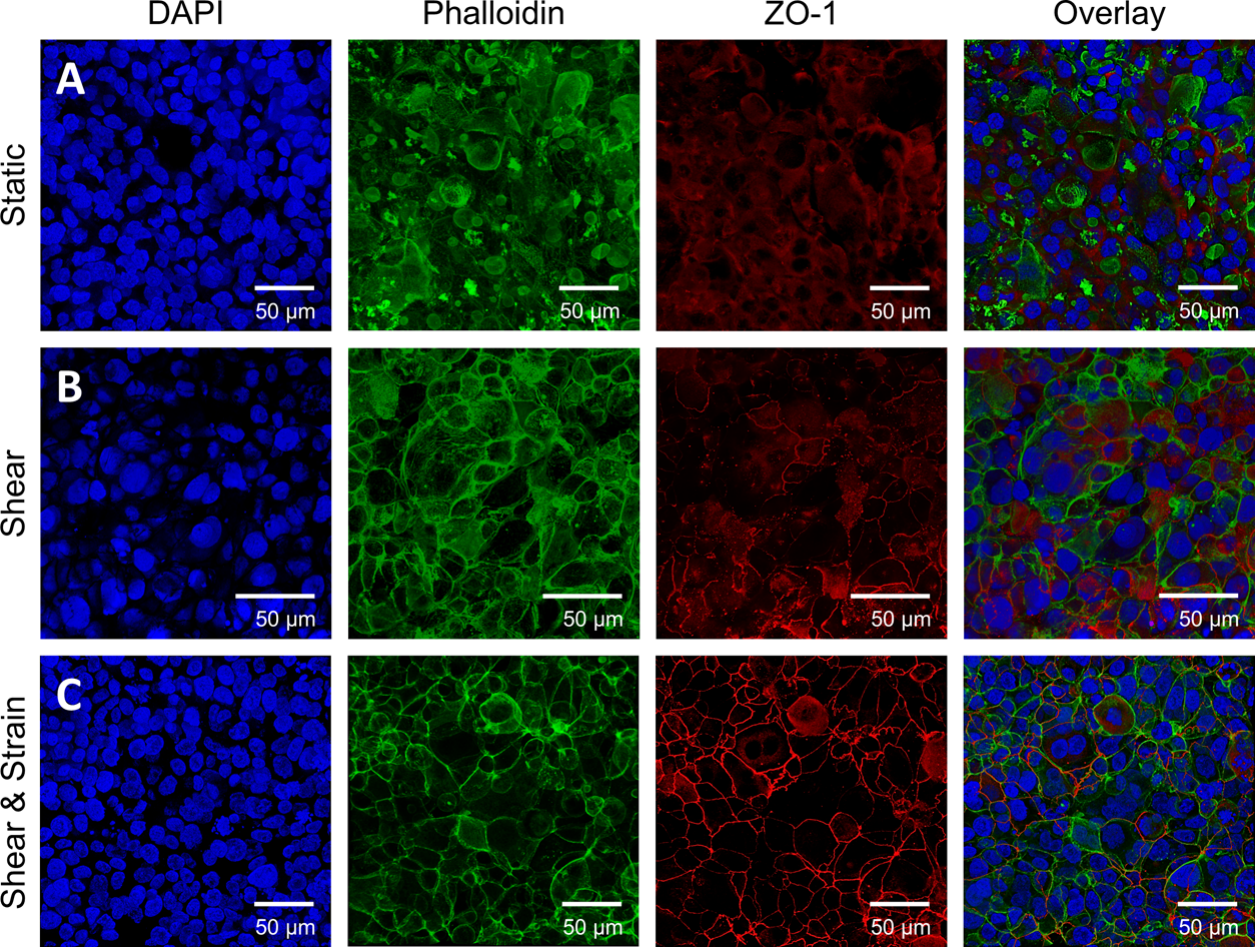
Analysis of actin distribution and tight junction formation by confocal fluorescence microscopy images of NCI-H441 pulmonary epithelial cells of co-cultures on biofunctionalized electrospun PCL-sPEG-NCO:RGD meshes exposed to different cultivation protocols. (A) Static control cultured at the air–liquid interface. (B) NCI-H441 monolayer exposed to 6 d of 1 dyn/cm^2^ fluid shear stress within the bioreactor system. (C) NCI-H441 monolayer exposed to 6 d of 1 dyn/cm^2^ fluid shear stress within the bioreactor system and 3 h of constant breathing motion at a frequency of 0.17 Hz at day 9. *n* = 3.

*In vivo*, pulmonary alveolar epithelial cells are subjected to cyclic strain and stress from breathing as well as blood flow in adjacent capillaries.^42^ The response of these cells to mechanical stimuli is typically assessed in terms of actin distribution and tight junction formation. Figure 7 demonstrates confocal fluorescence microscopy images of the NCI-H441 monolayer side of the co-culture model at day 6 of cultivation for the three previously introduced cultivation protocols. Here, blue presents cell nuclei (DAPI), green shows the actin cytoskeleton (Phalloidin), and red displays tight junctions (ZO-1). Comparing the results of the static control (Figure 7A) to co-culture models exposed to either fluid shear or a combined mechanical stimulus of shear and cyclic strain (Figure 7C), an increase in tight junction formation can be observed in models that experienced mechanical stress. However, NCI-H441 static models at the air–liquid interface have been shown to develop stable tight junctions when cultivated for longer periods of 9–17 days.^44^ Here, the integral tight junction formation is enhanced when exposed to both fluid shear stress and cyclic distension generated by air pressure oscillation. Additionally, in terms of actin distribution, the results demonstrate F-actin relocation toward the cell periphery (Figure 7B,C). It is known that the proline-rich C-terminal of ZO-1 binds F-actin suggesting a direct interaction of tight junctions and actin cytoskeleton.^45^ The presence of ZO-1 also indicates the absence of epithelial to mesenchymal transition, which is reinforced by the low percentage (5–7%) of α-smooth muscle actin (αSMA) present in NCI-H441 cell layers provided in Figure S11. Considering that the formation of tight junctions is a vital physiological function of pulmonary alveolar epithelial cells, these results demonstrate that inducing physiological stimuli to *in vitro* models can enhance the barrier properties of the respective model tissue and bring it closer to its *in vivo* counterpart.^46^

#### Influence on Epithelial Surfactant Protein B Production

**Figure 8:**
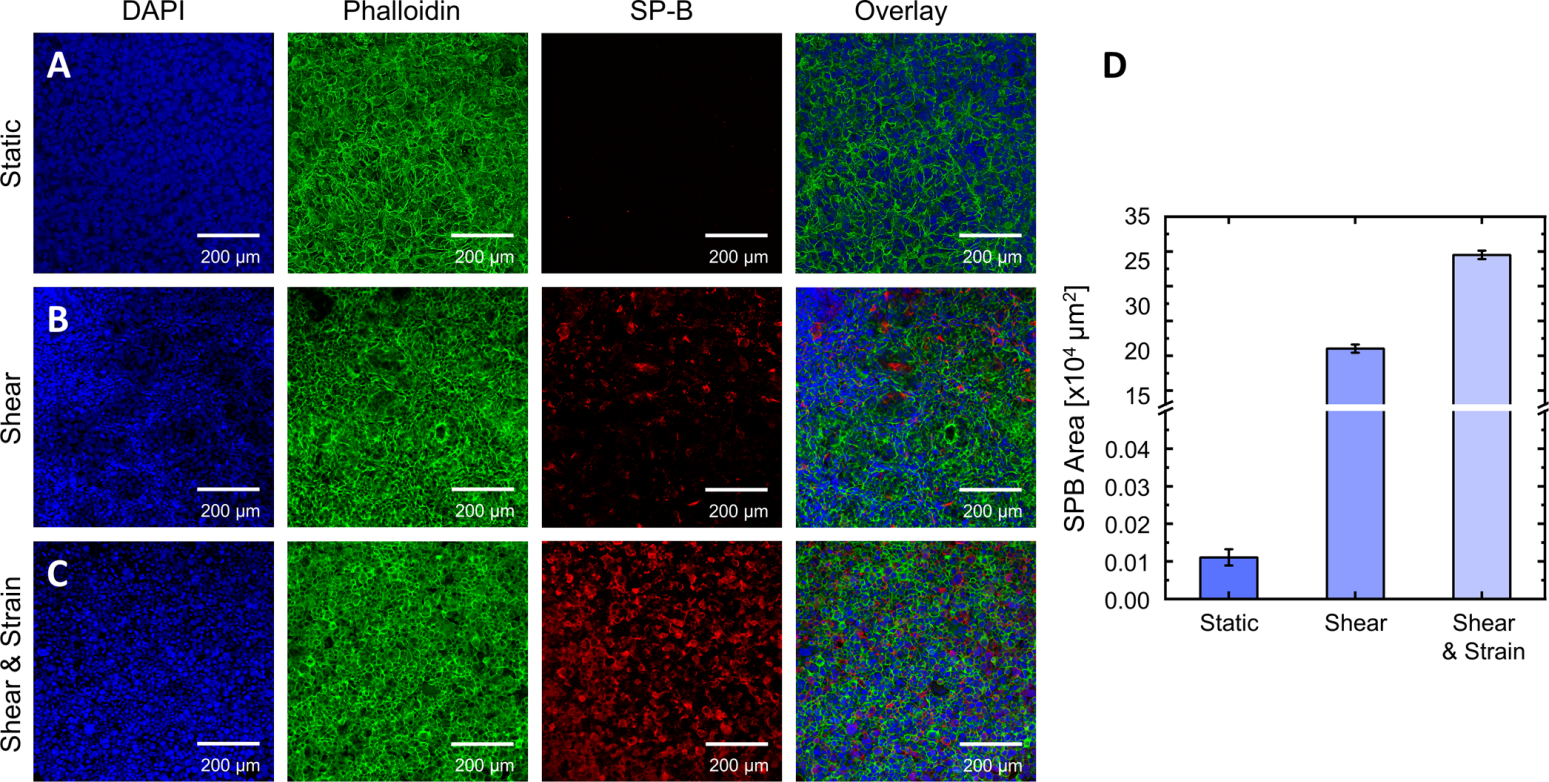
Analysis of surfactant protein B by confocal fluorescence microscopy images of NCI-H441 pulmonary epithelial cells of co-cultures on biofunctionalized electrospun PCL-sPEG-NCO:RGD meshes exposed to different cultivation protocols. (A) Static control cultured at the air–liquid interface. (B) NCI-H441 monolayer exposed to 6 d of 1 dyn/cm^2^ fluid shear stress within the bioreactor system. (C) NCI-H441 monolayer exposed to 6 d of 1 dyn/cm^2^ fluid shear stress within the bioreactor system and 3 h of constant breathing motion at a frequency of 0.17 Hz at day 9. (D) Analysis of surfactant protein B production in terms of traceable area. *n* = 3.

The human pulmonary adenocarcinoma NCI-H441 cell line is used to represent the alveolar epithelial cells as they display similar properties to type II pulmonary alveolar cells and can produce surfactant proteins SP-A and SP-B. The produced surfactant is a thin layer of phospholipids and proteins (SP-A, SP-B, SP-C, SP-D) present on the epithelial cell layer at the air–liquid interface. This 200 nm thin layer is known to have biophysical and immune functions, where it prevents lung alveoli collapse by reducing surface tension.^47^ Surfactant production is an intracellular process that includes the packing of lipids and proteins into lamellar bodies. The lamellar bodies are later released into the extracellular region, which leads to surfactant layer formation. Mechanical stimulations promote type II cell differentiation which is accompanied by increased SP-B production.^48^ Therefore, the expression of SP-B under the absence and presence of mechanical deformations at the air–liquid interface was investigated. Under static conditions, a very low amount of SP-B was detected, as seen in Figure 8A. Although NCI-H441 cells are known to express SP-B, our static models display low levels of SP-B expression, possibly due to an insufficient culture period.^49,50^ However, an increase in SP-B was observed on exposure to shear stress compared to static models (Figure 8B). Since the epithelial cell layer never directly experiences fluid shear stress, the question arises whether the elevated levels of surfactant production are due to minor mesh oscillations despite the dampening system or to endothelial-epithelial cell signaling. Therefore, we conducted a control experiment in which an epithelial NCI-H441 monolayer was cultured under continuous medium perfusion in the absence of endothelial cells. The results, which are depicted in Figure S12, show that NCI-H441 monolayers produce decreased amounts of SP-B compared to HPMEC-NCI-H441-co-culture models under similar cultivation conditions. Therefore, our control indicates cell-cell communication as the cause for elevated SP-B production in co-culture models exposed to shear stress. Indeed, corresponding literature suggests that this phenomenon might possibly be related to angiocrine signaling which is enhanced under shear stress close to physiological conditions.^51−53^ In this context, previous studies could demonstrate an interplay between alveolar epithelial and endothelial cells, where endothelial nitric oxide (NO) release influences alveolar development and up-regulates surfactant production by alveolar type II cells.^54^ However, this discussion is hypothetical in nature and requires additional experiments to identify a definitive mechanism. Compared to models exposed to shear stress, the SP-B was further increased on exposure to both shear and 3 h of cyclic strain followed by a 24 h recovery period under shear conditions (Figure 8C). This behavior is similar to previous studies, where cyclic strain is observed to significantly enhance surfactant protein production.^55,56^ This increase in SP-B is in accordance with its physiological function, where mechanical strain triggers an elevated expression of SP-B, which in turn protects the cells from its adverse effects. SP-B is integral in lowering surface tension to induce normal breathing and prevent pulmonary alveolar collapse.^57^ Therefore, the presence of both mechanical shear and strain at the air–liquid interface enhances SP-B expression that supports and maintains physiological pulmonary alveolar functions.

#### Influence on Endothelial Morphology

**Figure 9:**
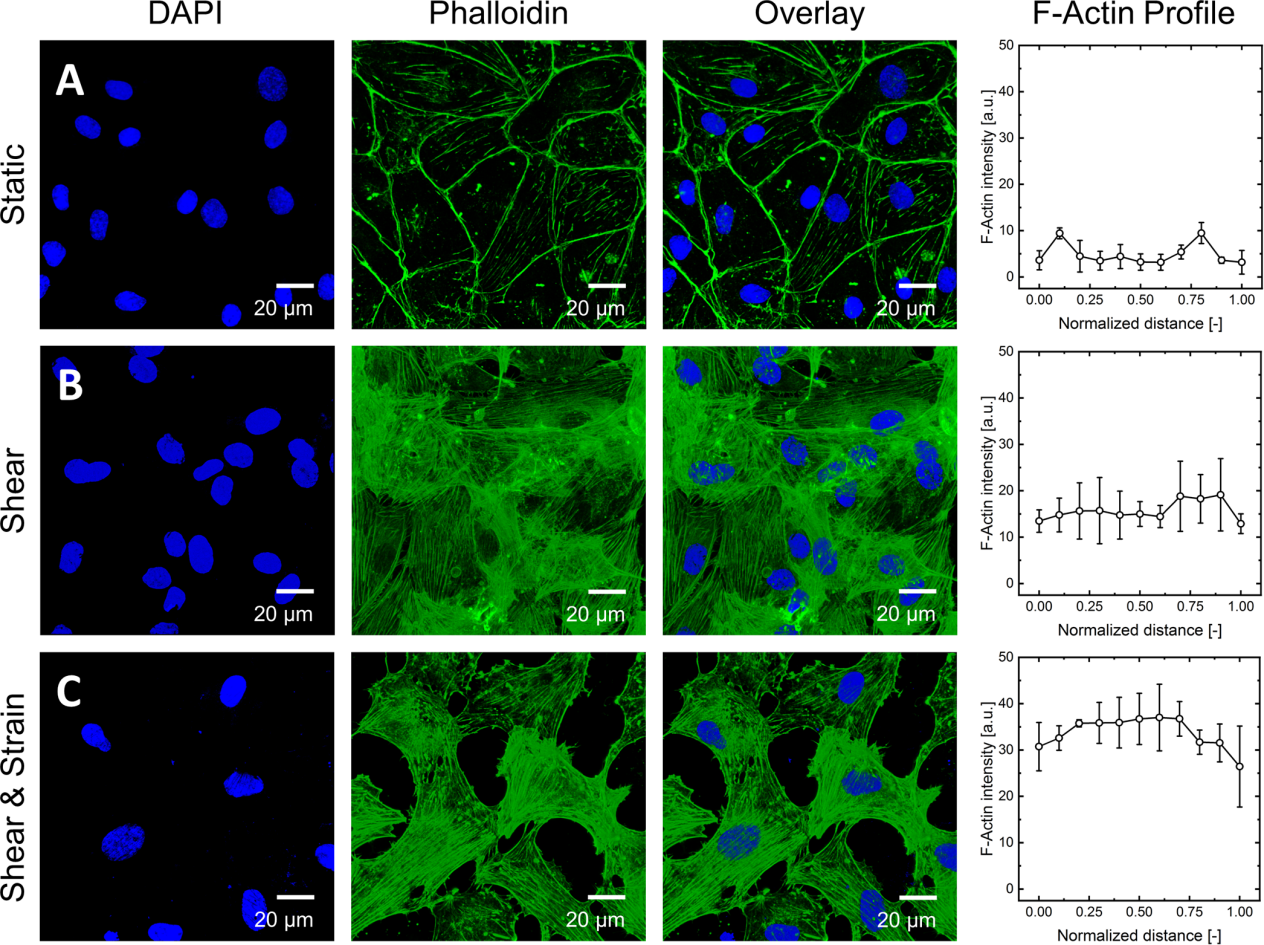
Analysis of actin distribution by confocal fluorescence microscopy images of HPMEC pulmonary endothelial cells of co-cultures on biofunctionalized electrospun PCL-sPEG-NCO:RGD meshes exposed to different cultivation protocols. (A) Static control cultured at the air–liquid interface. (B) HPMEC monolayer exposed to 6 d of 1 dyn/cm^2^ fluid shear stress within the bioreactor system. (C) HPMEC monolayer exposed to 6 d of 1 dyn/cm^2^ fluid shear stress within the bioreactor system and 3 h of constant breathing motion at a frequency of 0.17 Hz at day 9. *n* = 25.

In addition to the epithelial layers at the air–liquid interface, the endothelial cells were co-cultured on the opposite side and exposed to constant medium flow. It is known that endothelial cells are sensitive to shear stress in the physiological range (0.1–80 dyn/cm^2^).^7^ Under physiological stress, the endothelial cells undergo morphological changes in terms of cytoskeleton rearrangement and elongation. F-actin filaments can organize into cytoskeletal structures including membrane skeleton, cortical actin rim, and stress fibers. The membrane skeleton and cortical actin rim are situated close to the cell membrane, but the stress fibers extend across the cell body.^58^ The endothelial cells were exposed to a shear stress of 1 dyn/cm^2^ as well as both 1 dyn/cm^2^ shear and 1 mbar oscillatory strain at 0.17 Hz followed by an analysis of their actin cytoskeleton. In the absence of mechanical deformation (static), the endothelial cells revealed a distinct arrangement of actin on the periphery with few or no stress fibers across the cell body (Figure 9A). However, in the presence of shear, the endothelial cells displayed a characteristic distribution of stress fibers across the entire cell body (Figure 9B). Endothelial cells are known to respond to shear by increased, and thicker actin stress fiber formation, as well as cell elongation.^59^ This phenomenon is observed in both cases of shear alone as well as shear and strain (Figure 9C), where endothelial cells display thick stress fibers throughout the cell body. The actin profile distribution is depicted in the graphs of Figure 9, where actin intensity peaks at the cell periphery in static models, compared to an overall increased intensity throughout the cell body in the presence of mechanical stimulation. Moreover, in the case of a combined stimulation by shear and cyclic strain, the endothelial monolayers develop gaps between cells. This effect may be attributed to the orientation of the endothelial cells under cyclic strain, which has also been observed in endothelial layers cultured on fibronectin-coated silicon chambers that orient perpendicular to biaxial and uniaxial stretch.^60^ However, the influence of cyclic strain and stress on endothelial layers cultured on electrospun meshes has not yet been thoroughly investigated. In this context, Yang et al. could demonstrate that strains as low as 10% resulted in the realignment of 12 wt % PCL nanofibers in the direction of load.^61^ The cyclic strain induced for 3 h could therefore lead to fiber realignment and pore size enhancement which further affects the distribution of the sensitive primary endothelial cells leading to gaps in the confluent monolayer. Further analysis of structural changes in strained nanofiber meshes needs to be conducted to understand their role in cell adhesion and distribution.

## Conclusions

In this study, a bioreactor system was designed to induce mechanical forces, including fluid shear stress and triaxial periodic strain on *in vitro* co-culture models representing a functional pulmonary alveolar capillary barrier unit located at the air–liquid interface. The bioreactor allows the use of RGD-functionalized electrospun PCL-sPEG-NCO:RGD meshes closely mimicking the topology and function of a natural basement membrane, while simultaneously enabling the cultivation of pulmonary NCI-H441 epithelial and HPMEC endothelial cells at physiological levels of mechanical stimulation imitating blood flow and breathing motion. The incorporation of a fluid-dampening system eliminates undesired pulsatile flow originating from the peristaltic pump and allows independent adjustment of medium flow rate and periodic strain generated by air pressure oscillation. Comparative investigations could demonstrate an increased tight junction formation, an elevated surfactant protein B production, and a rearrangement of the actin cytoskeleton on both the epithelial as well as endothelial side of co-culture models exposed to mechanical stimulation. In this context, a combined mechanical stimulus involving fluid shear stress and periodic triaxial strain proved to be most effective in developing distinct phenotypical cellular features. The investigation of α-smooth muscle actin additionally revealed that *in vitro* models cultured on electrospun PCL-sPEG-NCO:RGD fiber meshes exhibit negligible epithelial to mesenchymal transition independent of the absence or presence of stimuli. Current limitations of the bioreactor system are the sensitivity regarding the pressure distribution, which causes labor-intensive manual operation and complicates the exposure of *in vitro* models to cyclic stretch for elongated time periods (>8 h) due to the necessity of surveillance. However, this challenge could be tackled by introducing a fully automated temperature and pressure control system that can read and adjust the corresponding parameters in a feedback loop in case of minor fluctuations. In summary, our study demonstrates the feasibility to substitute commonly applied microfluidic chips by 3D-printed reusable bioreactor systems and highlights the importance of introducing dynamic physiological conditions to the microenvironment of *in vitro* models for inducing phenotypical behavior. The application of ECM-mimicking scaffolds in combination with dynamic bioreactor systems could therefore lead to a more accurate representation of tissue sections in fundamental biological research, the study of aerosol toxicity, or drug tests.

## Experimental Section

### Device Fabrication

The polymeric components of the bioreactor, as well as the PDMS channel mold were fabricated from a heat-resistant acrylate-based photopolymer (RGD525, Stratasys) using the Objet Eden 260 V polyjet 3D printer (Stratasys). During the manufacturing process, overhangs and internal structures were stabilized by a support material (SUP705, Stratasys) which was removed after printing by mechanical ablation and dissolution in 1 M sodium hydroxide solution under constant stirring (370 rpm, 30 °C, 12 h). The components were rinsed in DI water and dried in ambient air. The three-way tubing connectors were 3D-printed using a Form 3 stereo-lithography printer (Formlabs) in combination with the corresponding high-temperature photopolymer (FLHTAM02, Formlabs). After fabrication, the tubing connectors were immersed in isopropanol (99.5%, Sigma-Aldrich, I9516) and were cleaned in an ultrasonic bath for 5 min. The reactor base plate was manufactured from a 2 mm thick aluminum plate using a milling machine (Trochoidal Performance Cutting, VollHartMetall).

### PDMS Channel Fabrication

For the fabrication of flow channels, polydimethylsiloxane (DowCorning, Sylgrad 184, 1,673,921) was mixed at a 10:1 ratio with the respective curing agent and was rotated at 15 rpm for 15 min to achieve a homogeneous solution. The mixture was poured into the respective molds, which in turn were placed for 1.5 h in a desiccator to remove entrapped air. The bubble-free solution was subsequently cured at 55 °C overnight before being removed from the mold using a scalpel. Finally, the PDMS channels were placed into an isopropanol-containing reagent tube and were cleaned by ultrasonication. Before being applied in the assembly, PDMS channels were incubated at 55 °C overnight to remove residual isopropanol from the PDMS matrix. A detailed drawing of the mold is provided in Figure S13.

### Bioreactor System Components and Assembly

The fluid-dampening system was assembled from 25 mL glass bottles (Schott, Carl Roth, A356.1), in-house fabricated three-way tubing connectors, poly(tetrafluoroethylene) (PTFE) sealing tape (Sigma-Aldrich, Z104388), and GL25 DURAN plastic screw caps with aperture (VWR, 201-1923). The PTFE tape was wrapped around the tubing connector base plate and the thread of the bottles to provide airtight sealing of the containers. Each dampening bottle was additionally equipped with an 8 cm long ROTILABO silicon tubing (2.0 mm ID, Carl Roth, 9559.1) that contained a clamp and a 0.2 μm PTFE sterile filter (Cole-Parmer, EW-15945-42) for pressure equalization. The aeration reservoir was built up from a 100 mL glass bottle (Schott, Carl Roth, KCN6.1), a GL45 silicon sealing ring (bbi-biotech, bbi-44130451), a stainless-steel tubing connector (bbi-biotech, bbi-44130406) and a GL45 DURAN plastic screw cap with aperture (VWR, 201-1925). Sterile media aeration was guaranteed by equipping the air inlet with a 0.2 μm PTFE sterile filter (Cole-Parmer). The bottles were interconnected using ROTILABO silicon tubing (2.0 mm ID, Carl Roth), TAAT PharMed pump tubing (2.06 mm ID, Tubing International,070539-15), and heat-resistant tube-to-tube connectors (Colder Products Company, SchellenShop, 2000000095464). The tubing system destined for oscillatory air supply was assembled from a 0.2 μm PTFE sterile filter (Cole-Parmer), a 100 mL glass bottle (Schott, Carl Roth), a GL45 silicon sealing ring (bbi-biotech), a stainless-steel tubing connector (bbi-biotech) as well as a GL45 DURAN plastic screw cap with aperture (VWR) and was interconnected by ROTILABO silicon tubing (2.0 mm ID, Carl Roth). The fully assembled medium circulation system was finally steam autoclaved (SystecTM VX-95) at 121 °C for 20 min together with the rest of the bioreactor components, including the 3D-printed parts, the aluminum base plate, the PDMS channel, the sealing ring (11 mm × 2 mm, Silicone), the microscopy glass (VWR, 631-0153), the Axygen polypropylene sealing (60 μm, Corning, PCR-TS) as well as the screws and nuts. While an ELVEFLOW OB1 pressure controller system was used for the generation of a sinusoidal air pressure profile, an ISMATEC 2-channel peristaltic pump was operated to generate a continuous medium supply.

### Shear Stress Simulation

The shear stress on the membrane area was simulated in COMSOL Multiphysics (vers. 5.5 COMSOL Inc., Burlington, MA) with the single-phase flow module. Water material properties were used as preset (viscosity, 1.002 mPas; temperature 293.15 K; and density, 998 kg m^–3^) to simulate five different volume flows (2.5, 5.0, 7.5, 10.0, and 12.5 mL/min). The shear stress distribution was evaluated by drawing samples from a uniform 1000 × 1000 rectangular grid in the flow direction.

### Preparation of Biofunctionalized Electrospun Meshes

Biofunctionalized nanofibrous meshes were fabricated in a one-step electrospinning process. To achieve 8.5 wt % solution, polycaprolactone (PCL) Mn 80.000 (Sigma-Aldrich, 440744) pellets were weighed and dissolved overnight in 1,1,1,3,3,3- Hexafluoro-2-propanol (HFIP) (Sigma-Aldrich, 105228). Functionalization was performed with RGD (Arg-Gly-Asp) peptides (Bachem, 4030602.0025) and 6-armed star-shaped isocyanate-terminated polyethylene glycol (sPEG-NCO).^39^ In brief, 1.5 wt % sPEG-NCO was dissolved in 50 μL of tetrahydrofuran (THF) (VWR, 348450010) and 10 μL of dimethyl sulfoxide (DMSO) (VWR, 23486.297) to which RGD was added to achieve a 5:1 molar ratio (sPEG-NCO:RGD). This mixture was stirred for 20 min and later added to the PCL solution. To further increase solution conductivity, 10 μL of 1% trifluoroacetic acid (TFA) (Sigma-Aldrich, T6508) was added. This mixture was used to electrospin functionalized nonwoven meshes. The earthed spinneret comprised a syringe with a flat-tipped 27-gauge needle (Braun, 8915992) filled with the described polymer solution. Nonwoven fiber meshes were achieved when the induced electric field was high enough to overcome the droplet’s surface tension and promote elongation of the Taylor cone. The meshes were collected on aluminum foil located on a metal plate (20 cm × 20 cm) connected to a high-voltage supply. The optimized spinning parameters included 21 kV, 15 cm (distance between collector and spinneret), and 0.75 mL/h to obtain homogeneous nanofibrous meshes. The electrospinning duration was kept constant at 3 min for 10 μm thin meshes.

### Characterization of Fiber Morphology

Scanning Electron Micrograph (SEM) images of the electrospun meshes were obtained using S-4800 ultrahigh-resolution SEM (HITACHI, Japan). Before imaging, the meshes were prepared by sputtering a 10 nm layer of Au/Pd using Leica EM ACE600. Images were taken at a working distance of 10–15 mm and an accelerating voltage of 20 kV. The SEM images were analyzed using the diameter plugin of ImageJ to obtain fiber diameter and pore area of the meshes. For this purpose, the images were converted to an 8-bit format where the desired features were extracted *via* threshold function. These images were then analyzed using the diameter plugin. Static contact angle measurements were conducted by applying a sessile drop method using the Krüss Drop shape analyzer (DSA100). An inbuilt software DSA4 was used to measure the contact angle of 3 μL distilled water droplets placed on the nonwoven meshes.

### Tensile Measurements

Deformation properties of the electrospun meshes were investigated by applying force corresponding to 20 N cell load in an AllroundLine, Zwick Roell (Germany) tensile tester. The electrospun meshes were transferred to an aluminum frame of 3 cm (length) × 1.5 cm (width). The frames holding the electrospun meshes were inserted into clamps, and the edges of the aluminum frame were cut to exclude their influence. Young’s modulus was obtained by calculating the slope of stress over strain within a 10% linear strain region. Additionally, a cyclic 15% strain at 30 cycles was applied to test the characteristics of the mesh resembling breathing deformations. The measurements were conducted in the wet state using phosphate-buffered saline (1× PBS).

### Cell Maintenance

The primary human pulmonary microvascular endothelial cells (HPMEC, Promocell, C-12281) were maintained and cultured in 2 wt % gelatin-coated culture (Sigma-Aldrich, G9391) flasks using microvascular endothelial growth media (EGM) (Promocell, C-22120). Epithelial human lung adenocarcinoma cell line (NCI-H441, ATCC, ATCC-HTB-174) was cultured in RPMI-1640 (Thermofisher Scientific, 21875) supplemented with 1% penicillin/streptomycin (Thermofisher Scientific, 15140) and 10% fetal bovine serum (FBS, BH Biowest, 5181). The environmental conditions for both cell types were 37 °C and 5% CO_2_. When cells achieved 85% confluency, they were either subcultured or used for experiments. While HPMEC were not subcultured above passage 4, NCI-H441 cells were used until passage 20 for experiments.

### Cell Seeding

The nanofibrous meshes were placed onto a custom 3D-printed transwell insert fabricated in-house using VeroClear material in combination with an Objet Eden 260V polyjet 3D printer (Stratasys). The meshes were immersed in PBS (Lonza, BEBP17-516Q) before carefully being removed from the collecting aluminum foils using tweezers. The meshes were then immersed in 70 vol % ethanol (BioUltra, Sigma-Aldrich, 51,976) for sterilization, placed in 1× PBS, and immobilized onto the inserts as shown in our previous publication.^2^ The fixed membranes were washed thrice with 1× PBS for 5 min each and were then ultraviolet (UV) sterilized for 30 min on each side. For cell seeding, a 60 μL cell suspension droplet of 8.5 × 10^4^ HPMEC in EGM is pipetted onto a lid of a 12-well microtiter plate. The insert holding the electrospun mesh is inverted and placed into the corresponding well. The microtiter plate is closed to establish contact between the cell suspension droplet on the lid and the mesh. The inserts are then incubated for 2 h at 37 °C and 5% CO_2_. This process step was followed by re-inverting the insert in a 24-well plate with the addition of 1000 μL of EGM in the basal compartment and 250 μL in the apical compartment. The following day, 250 μL of 1.5 × 10^5^ NCI-H441 cell suspension in RPMI-1640 medium was added to the apical side.^2^ The whole process of model fabrication is depicted in Figure S14 as a series of photographs.

### Static and Dynamic Cell Cultivation

Following the seeding procedure, the pulmonary alveolar capillary barrier models were cultured for 4 days under static conditions to achieve monolayer confluency, where medium exchanges were conducted on alternate days, including 1000 μL of endothelial growth medium in the basal compartment along with 250 μL of RPMI supplemented with 1 μM dexamethasone (Sigma-Aldrich, D4902) in the apical compartment. At day 4, the medium was aspirated from co-culture models destined for control purposes and 500 μL of EGM:RPMI-1640 (50:50) mixture supplemented with 1 μM dexamethasone was added to the basal compartment. The static control models were maintained for another 6 days at an air–liquid interface with medium exchanges performed every second day before being fixated in paraformaldehyde (PanReac Applichem, A3813). Samples destined for exposure to either fluid shear stress or a combination of shear and oscillatory strain were transferred from the transwell insert to the bioreactor membrane mount. The bioreactor system was subsequently assembled and connected to the circulation system, which was pre-heated to 37 °C. The setup was placed into the incubator, where it was connected to a peristaltic pump, and the dampening bottle pressure was equalized by opening and closing the airway clamps. The pump speed was set to 0.5 mL/min flow rate for the initial 2 h of cultivation to accustom the endothelial cells to the introduction of shear stress. Subsequently, the flow rate was incrementally increased by 0.1 mL/min every 2 h until the desired flow rate of approximately 1.1 mL/min was achieved. While co-culture models destined for exposure to fluid shear stress were cultured dynamically for 6 d until fixation, models destined for a combination of shear and oscillatory strain were additionally exposed to 3 h of periodic strain on day 9 induced applying an ELVEFLOW OB1 pressure controller at a frequency 0.17 Hz and an amplitude of 1 mbar.

### Immunofluorescence

Treatment with 4 vol % paraformaldehyde (PanReac Applichem) at room temperature (RT) for 15 min was carried out to fix cells, followed by permeabilization with 0.1 vol % Triton-X100 (Sigma-Aldrich, T9284) for 10 min. The samples were further blocked for 60 min with a solution of 3 wt % bovine serum albumin (BSA) (Sigma-Aldrich, A2153) in 1× PBS. Surfactant protein B staining was achieved by the addition of primary antisurfactant protein B antibody (R&D systems, IC1420T) at 1:100 at 4 °C overnight. Tight junctions were stained by incubating the specimen with primary ZO-1 antibody (Thermofisher Scientific, 61-7300) at a concentration of 1:100 at 4 °C overnight. The next day, samples were treated with secondary goat anti-rabbit Alexa Fluor 594 conjugated IgG (Abcam, ab150080) at a concentration of 1:200 for 2 h at RT. Samples were stained for F-actin by incubating the samples in a Phalloidin iFluor 488 reagent (Abcam, ab176753) at a concentration of 1:1000 for 2 h to visualize actin cytoskeleton. α-Smooth muscle actin (αSMA) was stained applying primary anti-αSMA (Sigma-Aldrich, A5228) at a dilution of 1:100 overnight at 4 °C followed by a subsequent incubation in secondary anti-mouse Alexa Fluor 488 at a concentration of 1:200 for 2h at RT. This process step was followed by 1× PBS wash and the addition of DAPI (Molecular probes, D1306) at a concentration of 1:1000 to stain the nuclei, followed by a PBS washing step. The mentioned washing steps with 1× PBS were carried out thrice in sequential steps at 5 min of incubation. The stained samples were removed from the inserts and placed between two microscopy glass slides (VWR), followed by confocal laser scanning microscopy analysis (Leica SP8 Falcon, Leica, Germany).

### Image Analysis

Confocal microscope images were analyzed using ImageJ software to determine the surfactant protein B area, and the actin profile. The area of surfactant protein B was determined using *n* = 3 images acquired at random locations. The images were converted to 8-bit, followed by thresholding. The “analyze particle” function was applied to measure the SP-B area. F-Actin profile was measured for *n* = 25 cells at arbitrary positions. The images were converted to 8-bit, and the toolbox was applied to draw a line perpendicular to the major axis of the cell through the cell center. The intensity progression across this line was measured by selecting the option “plot profile” within analyze tab. The data points were then exported as a CSV file, and the data points were normalized.

### Statistic Analysis

All data analyzed are expressed as mean ± standard deviation unless stated otherwise. Three individual experiments were carried out for statistical analysis, electrospun mesh characterization, and membrane distention. The area of surfactant protein B was determined using *n* = 3 confocal microscopy images acquired at random locations on the respective *in vitro* models. F-Actin profile was measured for *n* = 25 cells at arbitrary locations in each of the respective *in vitro* models.

## Supporting Information Available

Pressure distribution and trouble shooting; cross-section of the bioreactors’ housing component (Figure S1); detail part drawing of the bioreactors’ housing component as top view (Figure S2); detail part drawing of the bioreactors’s housing component as cross-section in side view (Figure S3); simulations of fluid shear stress and flow rate (Figure S4); assembled cultivation setup (Figure S9); contact angle measurement (Figure S10); αSMA staining in NCI-H441 (Figure S11); DAPI and SP-B staining in NCI-H441 (Figure S12); detail part drawing of the PDMS-based flow channel mould (Figure S13); overview of the seeding process (Figure S14) (PDF)
Response of mounted fiber meshes to flow rate 12.5 mL/min without dampening (Video S5) (MP4)
Response of mounted fiber meshes to flow rate 12.5 mL/min with dampening (Video S6) (MP4)
Response of mounted fiber meshes to flow rate 20.0 mL/min without dampening (Video S7) (MP4)
Response of mounted fiber meshes to flow rate 20.0 mL/min with dampening (Video S8) (MP4)
