# Supplementary material for: Peptide-Functionalized Electrospun Meshes for the Physiological Cultivation of Pulmonary Alveolar Capillary Barrier Models in a 3D-Printed Micro-Bioreactor
Source: ACS Biomater Sci Eng. 2023 Jul 4;9(8):4878–92. doi: 10.1021/acsbiomaterials.3c00047 (PMC10428094; doi:10.1021/acsbiomaterials.3c00047)
Supplement: Supplementary file 1 — ab3c00047_si_001.pdf [file ab3c00047_si_001.pdf]

Supporting Information

Peptide-Functionalized Electrospun Meshes for the Physiological Cultivation of Pulmonary Alveolar Capillary Barrier Models in a 3D-Printed Micro-Bioreactor

Puja Jain,[†,‡] Sebastian B. Rauer,[†,¶] Daniel Felder,[‡] John Linkhorst,[¶] Martin Möller,[‡] Matthias Wessling,[*,¶,‡] and Smriti Singh[*,§]

†*contributed equally to this publication*

‡*DWI - Leibniz Institute for Interactive Materials, RWTH Aachen University, Aachen*

¶*Institute for Chemical Process Engineering, RWTH Aachen University, Aachen*

§*Max Planck Institute for Medical Research, Jahnstraße 29, 69120 Heidelberg, Germany*

E-mail: manuscripts.cvt@avt.rwth-aachen.de; smriti.singh@mr.mpg.de

Pressure distribution. When the temperature of a closed system containing a gas phase is elevated, the gas phase expands and causes an increase in system pressure. This phenomenon also happens inside the air-tight dampening bottles when placed inside the incubator and results in bottle pressures above the ambient value. When the pump is now activated, the medium flow causes a pressure increase in the high-pressure dampening bottle (HPDB) and a pressure decrease in the low-pressure dampening bottle (LPDB). However, since the pressures in both bottles are already significantly exceeding the ambient pressure, the absolute pressure value $|-p|$ in the LPDB never reaches the absolute value $|+p|$ of the HPDB. As a result, the hydrostatic pressure at the cultivation area exceeds ambient pressure, and the liquid level within the membrane mount rises.

This phenomenon can be prevented by pre-heating and venting the dampening bottles. The pre-heating to 37°C increases the temperature of the bottle's gas and liquid phase, while the subsequent venting procedure decreases the pressure back to its ambient value. However, depending on the accuracy of the temperature measurement device and the homogeneity of the heating process, minor temperature deviations remain after the pre-heating process. These temperature deviations can cause a time-dependent shift in bottle pressure as they equilibrate to 37°C within the incubator, resulting in hydrostatic pressure changes at the cultivation area. Since the hydrostatic pressure is required as the base value for the pressure oscillation to maintain a stable air-liquid interface, an equilibration period is necessary in case the pressures and temperatures are not monitored and controlled. We recommend an equilibration period of 1 day to ensure achieving equilibrium conditions. However, a more advanced temperature control system could render the equilibration time obsolete.

Trouble Shooting. Most of the challenges regarding the proposed bioreactor setup are related to the generation of the correct pressure distribution, which is heavily dependent on the air tightness of the dampening system and a correct heating protocol.

The air tightness of the dampening system can be tested before the experiment by heating the system to 37°C. If all clamps are closed, the increase in temperature leads to a visible rise of the medium level within the tubing system due to the expansion of the gas phase and the corresponding increase in gas pressure. In case both bottles are sufficiently air-tight, the medium level should reach equal distances measured from the dampening bottle caps. If the distances are significantly different, one of the dampening bottles leaks air and hence cannot build up the necessary gas phase pressure. In this case, the caps of the dampening bottles should be tightened again, or the PTFE sealing tape needs to be reinforced or exchanged. Another possible cause can be loose connections or leaky tubing.

After checking the air tightness of the system, it is important to ensure equal temperatures in all bottles before venting the system. If the bioreactor system is placed inside the incubator and the bottle temperatures significantly deviate from each other, the gas pressure changes occurring during the equilibration period will also differ leading to a shift in hydrostatic pressure at the cultivation area. This change in hydrostatic pressure can lead to changes in the liquid level and hence can cause flooding or drying up of the model. In case experimentation fails due to flooding or drying up, ensure that the heating procedure is performed for a proper duration and that all bottles exhibit close to identical temperatures. Other reasons could be weak pump cassette springs or the wear of pump tubing.

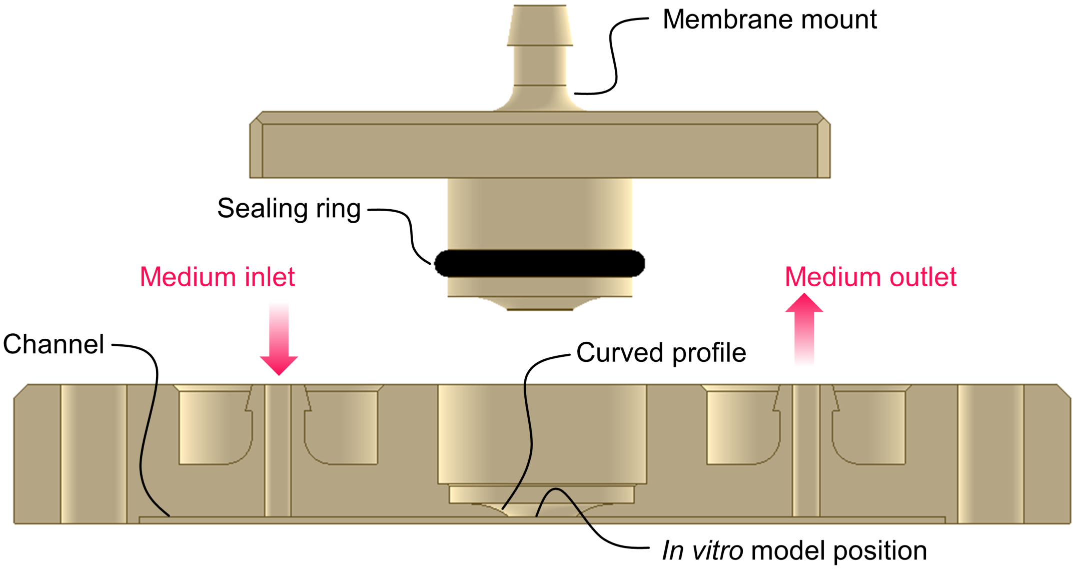

Fig. S1: Cross-section of the bioreactors' housing component. The scheme shows the membrane mount holding the electrospun mesh, the sealing ring preventing medium leakage and contamination, the medium in- and outlet, the cultivation channel housing the fluid flow and the position of the in vitro model. The chamber sealing located below the membrane mount displays a curved profile to maximize material thickness.

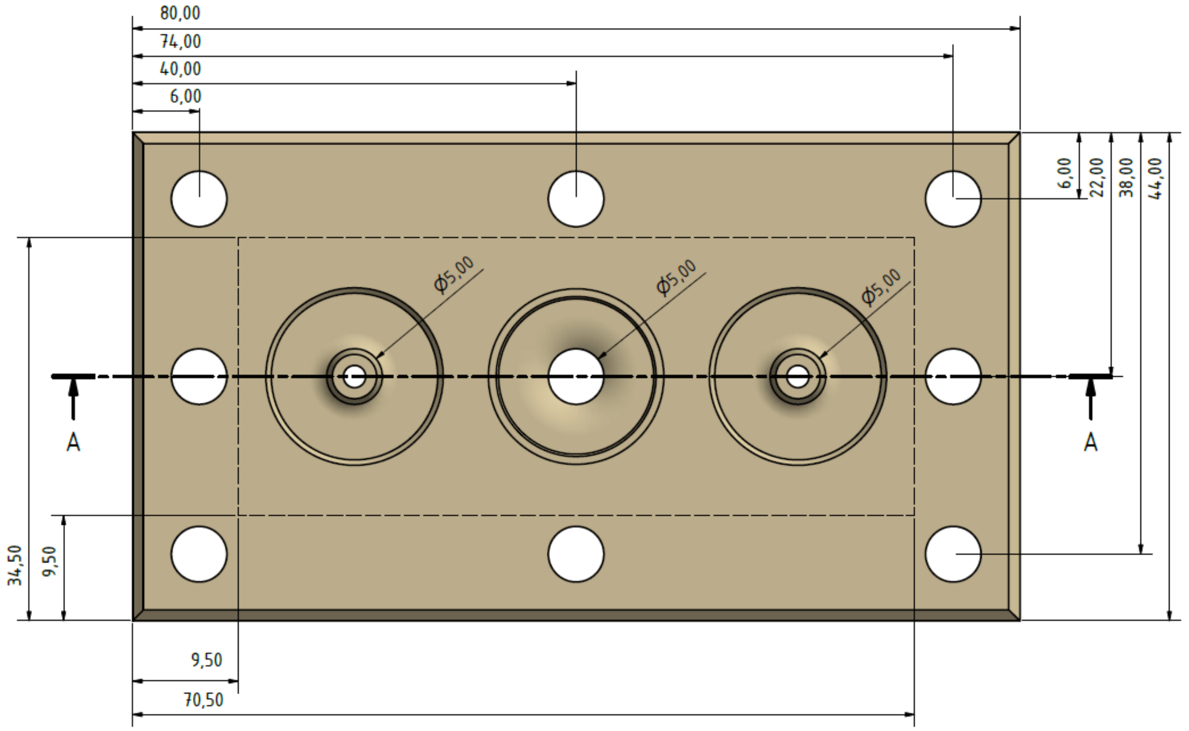

Fig. S2: Detail part drawing of the bioreactors' housing component as top view. The dashed line presents the rectangular groove introduced at the bottom of the housing component utilized for PDMS channel alignment and channel sealing. A-A indicates the cross-section cut for Figure S3. All units are given in millimeters.

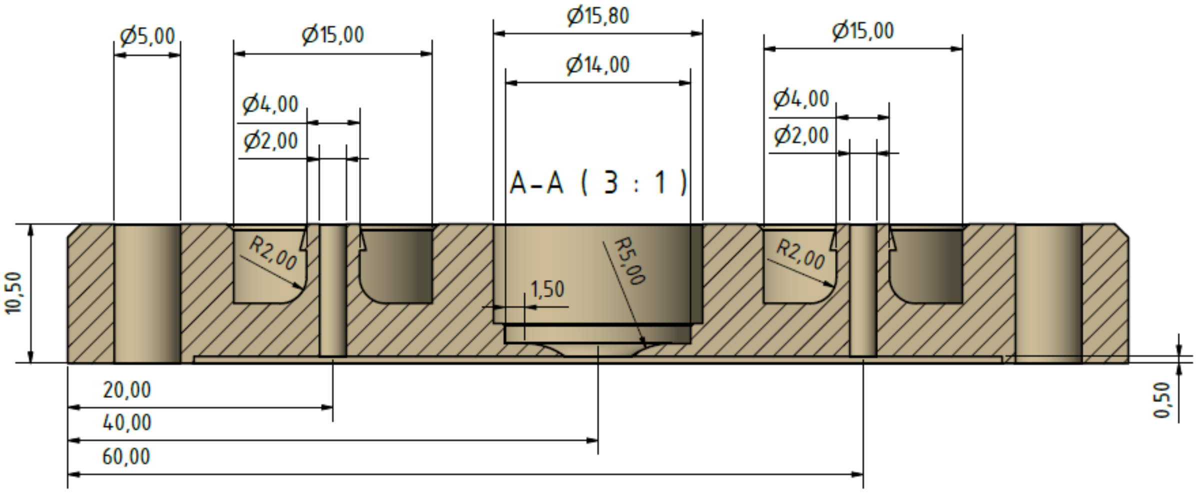

Fig. S3: Detail part drawing of the bioreactors' housing component as cross-section in side-view. Besides demonstrating the full set of housing dimensions, the image presents the height of the rectangular groove introduced at the bottom of the housing component utilized for PDMS channel alignment and channel sealing. All units are given in millimeters.

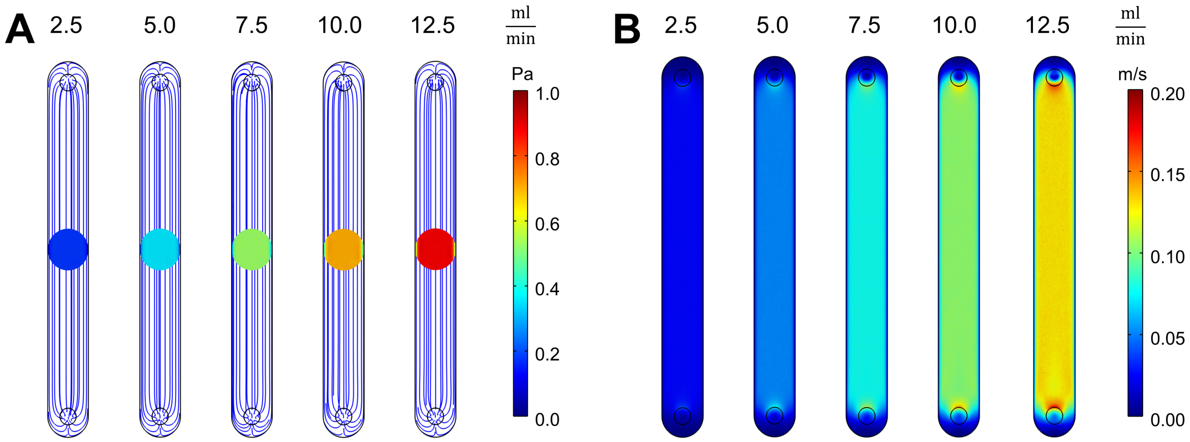

Fig. S4: Simulations of the shear stress (A) and flow rate (B) distribution within the perfusion channel of the bioreactor for different fluid velocities. The circular region located in the channel center indicates the cultivation area.

Video S5 - 12_5mlmin - Without Dampening: Electrospun mesh deflection caused by the pulsatile flow originating from a peristaltic pump at 12.5 ml/min in the absence of a dampening system.

Video S6 - 12_5mlmin - Dampening: Electrospun mesh deflection caused by the pulsatile flow originating from a peristaltic pump at 12.5 ml/min in the presence of a dampening system. The video showcases the absence of mesh deflections caused by a peristaltic pump below 12.5 ml/min.

Video S7 - 20_0mlmin - Without Dampening: Electrospun mesh deflection caused by the pulsatile flow originating from a peristaltic pump at 20.0 ml/min in the absence of a dampening system.

Video S8 - 20_0mlmin - Dampening: Electrospun mesh deflection caused by the pulsatile flow originating from a peristaltic pump at 20.0 ml/min in the presence of a dampening system. Onset of the efficiency decrease of the dampening system.

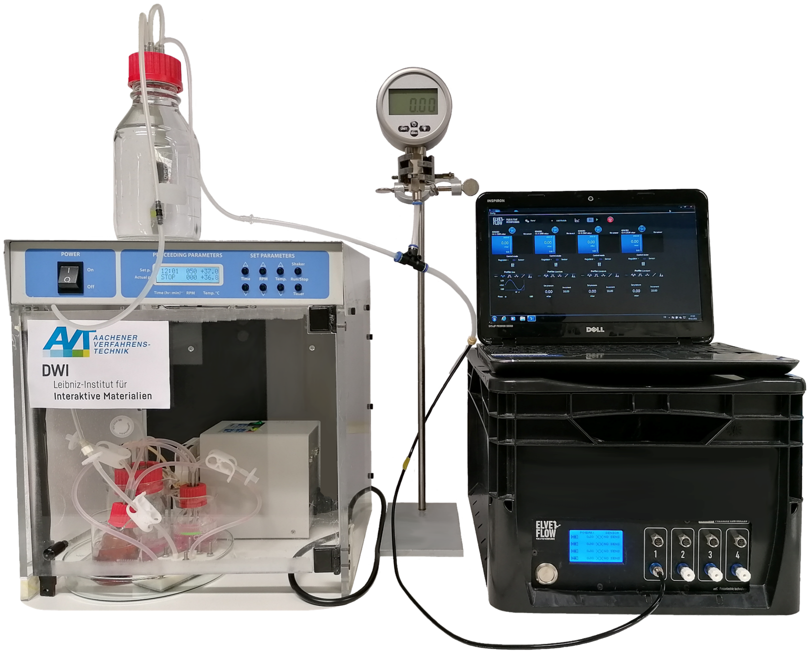

Fig. S9: Assembled cultivation setup including the bioreactor, two dampening bottles, an aeration bottle, and a peristaltic pump within a table incubator. The bioreactors' air compartment is connected to a pressure controller. The additional Schott flask protects the pressure controller from potential medium overflow, while the manometer is used as an additional control for air pressure.

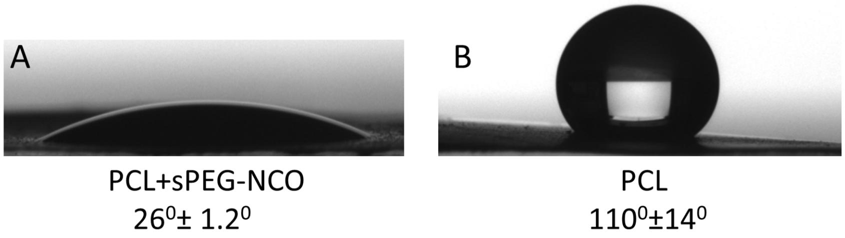

Fig. S10: Static contact angles were measured using sessile drop method to investigate the hydrophilic nature of the nanofibrous meshes. A) The water droplet spreads and displays a reduced angle of 26° ± 1.2° on meshes spun with sPEG-NCO which imparts hydrophilic properties to the PCL membranes. B) As control, pure PCL nanofibrous meshes display less spread and a higher contact angle of 110° ± 14° which is consistent with the inherent hydrophobic property of the PCL meshes.

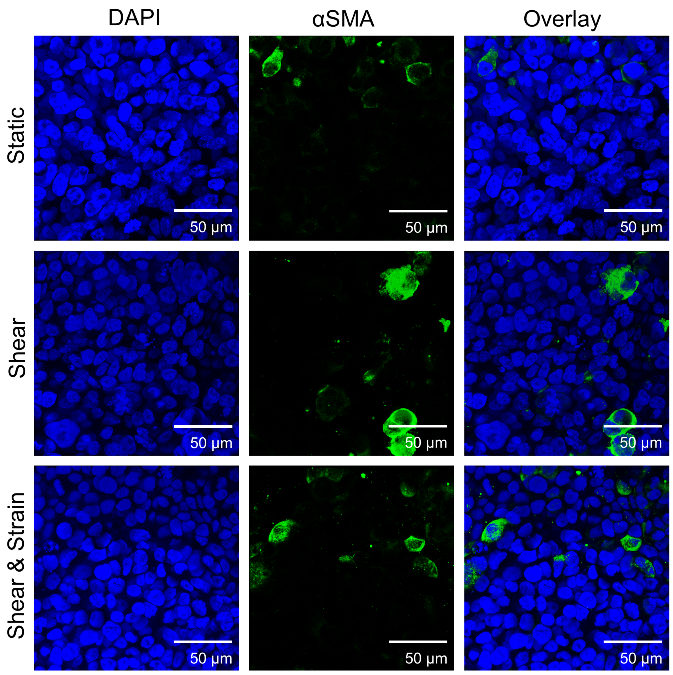

Fig. S11: Investigation of epithelial to mesenchymal transition by the study of α-smooth muscle actin (αSMA)in NCI-H441 at cultivation day 10 for different experimental procedures including static, exposure to shear stress, and a combination of shear stress and cyclic strain. In static, $5.4 \pm 1.5\%$ of NCI-H441 cells displayed αSMA, under shear stress, $5.6 \pm 1.1\%$ of the NCI-H441 cells displayed αSMA, and under a combination of shear stress and cyclic strain $6.9 \pm 1.4\%$ displayed αSMA. n = 5.

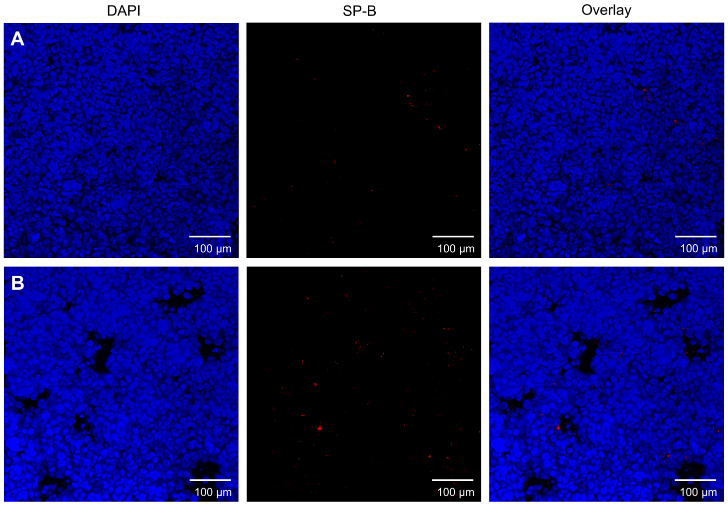

Fig. S12: Nucleus (DAPI) and surfactant protein B (SP-B) investigation on epithelial NCI-H441 cell monolayers exposed to 6 days of continuous physiological shear stress in the absence of endothelial cells. The results show a decreased amount of SP-B production in the absence of endothelial cells and the formation of randomly distributed holes in the epithelial monolayer. A) Fully-confluent region of the *in vitro* model. B) Perforated region of the *in vitro* model. Both regions A) and B) are located on the same *in vitro* model.

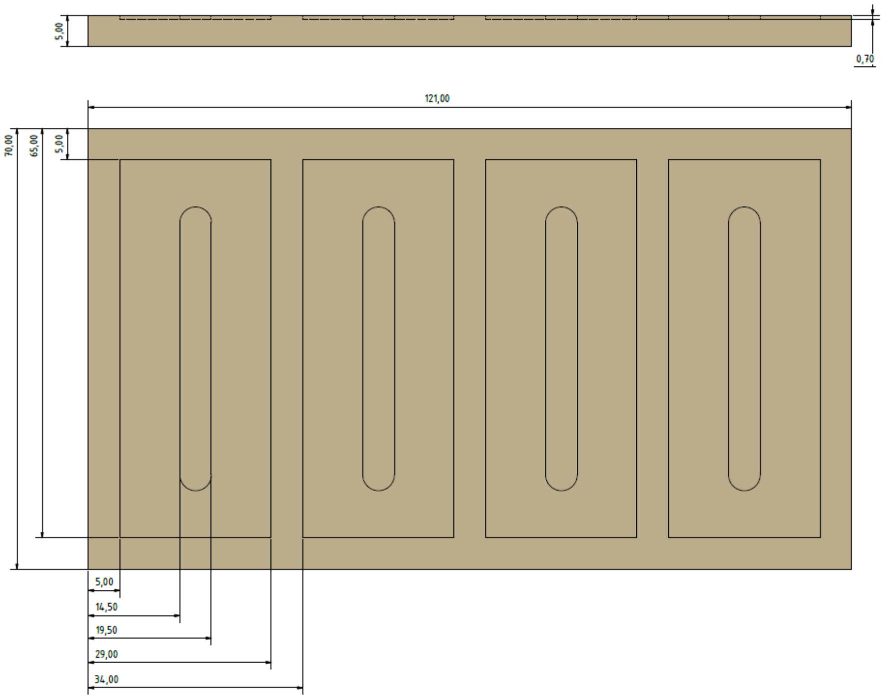

Fig. S13: Detail part drawing of the 3D-printed mould for the fabrication of the PDMS-based flow channels. To acquire even PDMS surfaces necessary for tight channel sealing the excess solution was scraped off the mould before being cured on a leveled surface. In early development, stages various height differences were tested. While smaller height differences between the groove and PDMS channel led to leakage due to insufficient force, larger height differences resulted in bioreactor failure due to the deformation of the housing component during operation. Considering that even most high-temperature 3D-printing materials tend to deform over time under continuous tension at elevated temperatures, the housing component was fabricated with an extensive height of 10.5 mm, while the base plate was manufactured from 2 mm thick aluminum.

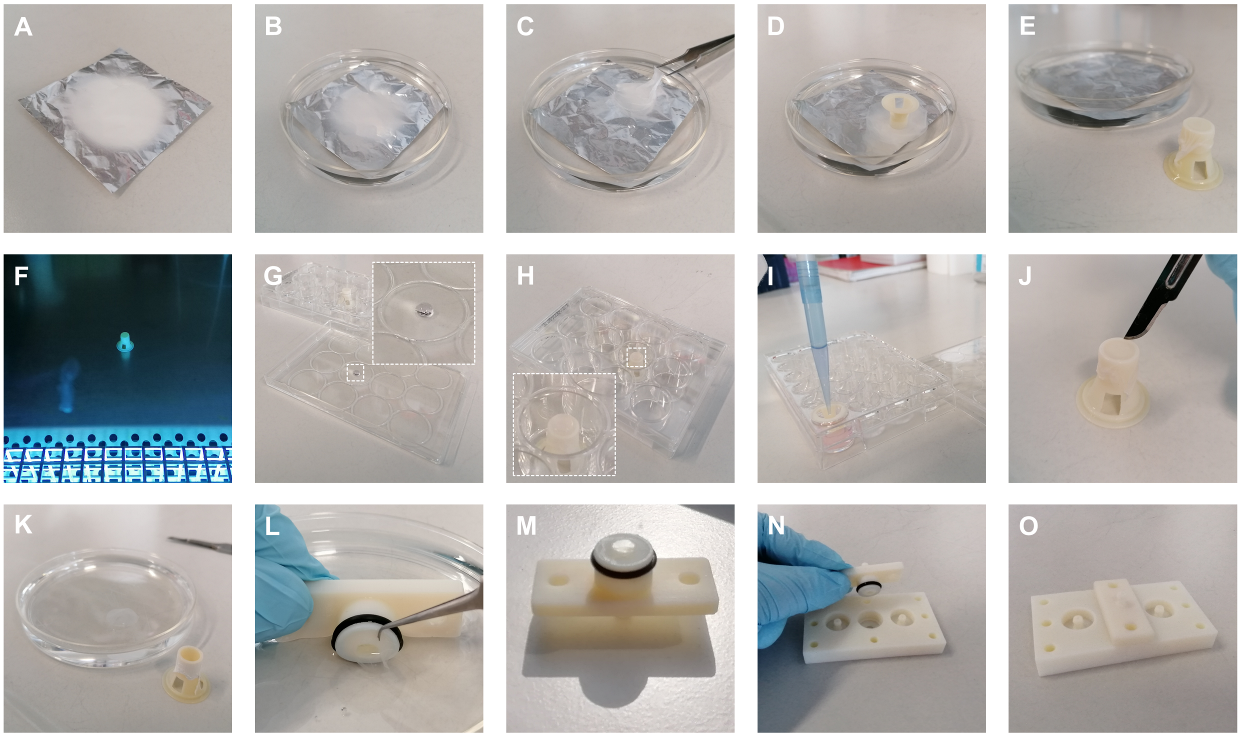

Fig. S14: Overview of the seeding process on PCL-sPEG-NCO:RGD fiber meshes, and transfer of cell layer-containing fiber meshes to the bioreactor. A) Aluminium foil holding a fiber mesh directly after electrospinning. B) Wetting of the fiber mesh in PBS (1X). C) Detachment of the fiber mesh using tweezers. D) Transfer of the fiber mesh to the 3D printed transwell insert. E) Transwell insert holding the fiber mesh. F) UV sterilization of the fiber mesh. G) – H) HPMEC seeding. A cell-containing medium droplet is pipetted onto the lid of a microtiter plate. The transwell insert holding the fiber mesh is inverted and placed into the corresponding microtiter plate. The microtiter plate is closed using the lid resulting in contact between the cell-containing droplet and the fiber mesh. The microtiter plate is subsequently incubated for 2h at 37°C enabling cell sedimentation and attachment. Finally, the transwell insert is inverted again and 1000 μL of medium are pipetted in the basal, and 200 μL in the apical compartment. I) After 1 day of HPMEC cultivation at 37°C, NCI-H441 cells are seeded into the apical compartment of the transwell insert. The NCI-H441/HPMEC model is cultured for 4 days at 37°C in RPMI1640/EGM medium to produce confluent cell layers. J) The confluent fiber mesh is cut loose using a scalpel. K) The fiber mesh is detached from the transwell insert in sterile PBS (1X). L) The membrane is transferred to the membrane mount of the cultivation chamber using tweezers. M) Membrane mount holding an electrospun fiber mesh. N) The membrane mount is plugged into the bioreactor. O) Fully assembled upper part of the bioreactor holding an electrospun membrane.
